# Hyaluronidase Impairs Neutrophil Function and Promotes Group B *Streptococcus* Invasion and Preterm Labor in Nonhuman Primates

**DOI:** 10.1128/mBio.03115-20

**Published:** 2021-01-05

**Authors:** Michelle Coleman, Blair Armistead, Austyn Orvis, Phoenicia Quach, Alyssa Brokaw, Claire Gendrin, Kavita Sharma, Jason Ogle, Sean Merillat, Matthew Dacanay, Tsung-Yen Wu, Jeff Munson, Audrey Baldessari, Jay Vornhagen, Anna Furuta, Shayla Nguyen, Kristina M. Adams Waldorf, Lakshmi Rajagopal

**Affiliations:** a Center for Global Infectious Disease Research, Seattle Children’s Research Institute, Seattle, Washington, USA; b Department of Global Health, University of Washington, Seattle, Washington, USA; c Washington National Primate Research Center, Seattle, Washington, USA; d Department of Obstetrics and Gynecology, University of Washington School of Medicine, Seattle, Washington, USA; e Department of Psychiatry and Behavioral Sciences, University of Washington, Seattle, Washington, USA; f Center for Innate Immunity and Immune Disease, University of Washington, Seattle, Washington, USA; g Sahlgrenska Academy, University of Gothenburg, Gothenburg, Sweden; h Department of Pediatrics, University of Washington, Seattle, Washington, USA; University of Pittsburgh School of Medicine

**Keywords:** group B streptococcus, hyaluronidase, immune evasion, neutrophils, pregnancy, preterm labor

## Abstract

Invasive bacterial infections during pregnancy are a major risk factor for preterm birth, stillbirth, and fetal injury. Group B streptococci (GBS) are Gram-positive bacteria that asymptomatically colonize the lower genital tract but infect the amniotic fluid and induce preterm birth or stillbirth. Experimental models that closely emulate human pregnancy are pivotal for the development of successful strategies to prevent these adverse pregnancy outcomes. Using a unique nonhuman primate model that mimics human pregnancy and informs temporal events surrounding amniotic cavity invasion and preterm labor, we show that the animals inoculated with hyaluronidase (HylB)-expressing GBS consistently exhibited microbial invasion into the amniotic cavity, fetal bacteremia, and preterm labor. Although delayed cytokine responses were observed at the maternal-fetal interface, increased prostaglandin and matrix metalloproteinase levels in these animals likely mediated preterm labor. HylB-proficient GBS dampened reactive oxygen species production and exhibited increased resistance to neutrophils compared to an isogenic mutant. Together, these findings demonstrate how a bacterial enzyme promotes GBS amniotic cavity invasion and preterm labor in a model that closely resembles human pregnancy.

## INTRODUCTION

Globally, invasive bacterial infections are a leading cause of stillbirth and preterm birth (1–3). Group B streptococci (GBS) or Streptococcus agalactiae are beta-hemolytic, Gram-positive bacteria that commonly exist as commensal organisms in the rectovaginal tracts of healthy adult women. However, GBS can be transmitted to neonates during birth or ascend into the uterus during pregnancy, resulting in fetal injury, stillbirth, preterm birth, or early neonatal infection (4–6). Recent reports have indicated that by conservative estimates, approximately 147,000 stillbirths and 3.5 million preterm births each year are attributed to GBS infections (7). Currently, there are no therapies to prevent preterm birth or stillbirth. Some countries have implemented protocols to screen women for GBS colonization in the third trimester of pregnancy and to subsequently administer antibiotics to GBS-positive women during labor and delivery (known as intrapartum antibiotic prophylaxis). Although these measures have decreased the incidence of neonatal GBS disease in the first week of life (8, 9), they fail to prevent adverse pregnancy outcomes that occur prior to labor and delivery that result in preterm births or stillbirths (8, 10–12).

Improved preventive therapies for GBS require a greater understanding of the complex interactions between pathogen and host. One host factor important for responses to microbial infection is hyaluronan (HA). HA is a major constituent of the host extracellular matrix and exists as a high-molecular-weight glucosaminoglycan polymer that assists in cell migration, cell-cell signaling, and responses to injury and infection (13, 14). During infection or injury, high-molecular-weight HA (HMW-HA) is degraded by host hyaluronidases or reactive oxygen species (ROS) to low-molecular-weight HA (LMW-HA; comprising HA tetramers or pentamers), which are proinflammatory in nature and mediate cytokine responses through signaling via Toll-like receptors (TLRs), such as TLR-2 and TLR-4 (15, 16). Interestingly, certain bacterial pathogens such as GBS also secrete a hyaluronidase enzyme (17, 18). The GBS hyaluronidase (HylB; encoded by the *hylB* gene) was identified in 1950 as an exolytic enzyme (19) that breaks down HA into disaccharide fragments (20). Recently, GBS HylB-generated HA disaccharides were shown to block TLR-2 and TLR-4 signaling in macrophages and dampen cytokine responses (21). Subsequently, we observed that clinical GBS isolates associated with women in preterm labor or neonatal infections exhibit increased hyaluronidase activity compared to commensal GBS isolates obtained from rectovaginal swabs of healthy women (22). We also noted that GBS hyaluronidase dampened uterine immune responses and postulated that these promoted ascending infection in a pregnant mouse model of GBS infection (22).

However, the pregnant mouse and other lower mammalian models exhibit dissimilarities to many aspects of human pregnancy, including differences in reproductive anatomy, placentation, onset of labor, and sensitivity to pathogens. In contrast, the closest animal model for studies related to human pregnancy is the pregnant nonhuman primate (NHP) (23–26). Similarities of NHPs to humans include reproductive anatomy, type and structure of placenta (hemomonochorial), number of fetuses (singleton), long gestational period (160 to 170 days), initiation of labor (hormonal control of parturition), sensitivity to pathogens, and the developmental timeline of the fetal lung and brain (25, 26). In the chronically catheterized pregnant NHP model (27, 28), we inoculate bacteria at the choriodecidual space, a site in the pregnant uterine mucosa between the uterine muscle and the placental membranes, where bacteria are thought to first encounter the maternal-fetal interface during ascending infection from the lower genital tract (2, 27).

Here, we used a nonhemolytic GBS strain (serotype V, GB37) that exhibits increased hyaluronidase activity (29) to address how hyaluronidase contributes to immune evasion, fetal injury, and preterm labor. We found that pregnant NHPs inoculated with GB37 exhibited rapid microbial invasion of the amniotic cavity, fetal bacteremia, and preterm labor in contrast to animals inoculated with the isogenic, hyaluronidase-deficient strain. Analyses of the cellular and biochemical events at the maternal-fetal interface revealed impaired host defenses that likely prompted the early onset of labor. Together, our studies show that a bacterial hyaluronidase promotes microbial invasion of the amniotic cavity and preterm labor in the nonhuman primate model.

## RESULTS

### HylB promotes adverse pregnancy outcomes in an NHP model of choriodecidual GBS infection.

To elucidate whether hyaluronidase promotes GBS infection and adverse outcomes during pregnancy, we used a chronically catheterized nonhuman primate (Macaca nemestrina) model that closely emulates human pregnancy and defines temporal events during amniotic cavity invasion and preterm labor (28). Ten animals received choriodecidual inoculations of 1 × 10^8^ to 3 × 10^8^ CFU of either hyaluronidase-proficient wild-type GBS (strain GB37, *n* = 5) or an isogenic hyaluronidase-deficient GBS (strain GB37Δ*hylB*, *n* = 5). Controls included NHPs that received saline (*n* = 6 total; *n* = 4 were described previously [27]). Adverse pregnancy outcomes, specifically preterm labor and/or microbial invasion of the amniotic cavity (Table 1) were the primary outcomes of our study. In GBS-inoculated animals, Cesarean section was performed at the onset of preterm labor, which was defined as progressive cervical dilation associated with increased and sustained uterine activity or at 3 days post-GBS inoculation if preterm labor did not occur, as described previously (28).

**TABLE 1:** Summary of pregnancy outcomes, cytokines, and prostaglandins in pregnant NHPs^*a*^

Outcome	Saline (*n* = 6)	GB37 (*n* = 5)	GB37Δ*hylB* (*n* = 5)	*P*		
				Saline vs GB37	GB37 vs GB37Δ*hylB*	Saline vs GB37Δ*hylB*
Primary and composite outcomes, no. (%)						
Adverse outcome^*b*^	0 (0)	5 (100)	1 (20)	**0.0009**	**0.02**	NS
Preterm labor^*c*^	0 (0)	4 (80)	1 (20)	**0.01**	0.1	NS
Microbial invasion of the amniotic cavity and fetal bacteremia	0 (0)	5 (100)	1 (20)	**0.0009**	**0.02**	NS
						
Peak contractions, AF cytokines, and prostaglandins, mean peak pg/ml (SEM)						
Hourly contraction area	1,326.7 (506.58)	5,606.0 (1,165.1)	3,087.4 (1,050.0)	**0.04**	NS	NS
IL-1β	31.61 (20.59)	1,754.8 (1,112.7)	597.65 (583.42)	0.09	NS	NS
TNF-α	26.13 (11.44)	574.58 (285.82)	97.58 (39.43)	0.07	NS	NS
IL-6	7,091.1 (2,642.2)	2,827.8 (1,331.3)	2,404.1 (1,356.5)	NS	NS	NS
IL-8	888.07 (321.53)	2,120.5 (1,383.3)	1,629.7 (1,498.2)	NS	NS	NS
PGE_2_	509.83 (247.84)	1,160.9 (647.40)	289.23 (116.69)	NS	NS	NS
PGF_2α_	424.31 (169.64)	1,808.1 (887.27)	164.93 (45.91)	NS	0.08	NS
						
Fetal cytokines						
IL-1β	0.80 (0.60)	2.152 (0.8584)	1.128 (0.8645)	NS	NS	NS
TNF-α	2.268 (1.127)	0.01 (0.0)	0.01 (0.0)	**0.04**	NS	**0.04**
IL-6	3.639 (1.852)	1,049.6 (722.10)	44.74 (41.58)	**0.049**	NS	NS
IL-8	574.06 (314.04)	2,648.8 (1,674.1)	2,576.2 (1,551.3)	NS	NS	NS

a The primary outcomes are shown as “number (%)” and were compared among groups using Barnard’s test. Amniotic fluid (AF) cytokines, prostaglandins and fetal plasma cytokines are expressed as mean peak (SEM) in pg/ml. Hourly contraction area is expressed in mm Hg × s/h. Cytokines (IL-1β, TNF-α, IL-6, and IL-8) and prostaglandins (PGE_2_ and PGF_2α_) were compared by using one-way ANOVA with Bonferroni’s correction. Statistical analyses were conducted using Intercooled STATA 8.2 for Windows 2000 (StataCorp) or SciStatCalc. *P* values of <0.05 are indicated in boldface. NS, *P* > 0.100.

b Adverse outcome is a composite metric representing preterm labor or microbial invasion of the amniotic cavity with fetal bacteremia.

c Preterm labor was defined as progressive cervical dilation associated with increased and sustained uterine activity.

**FIG 1:**
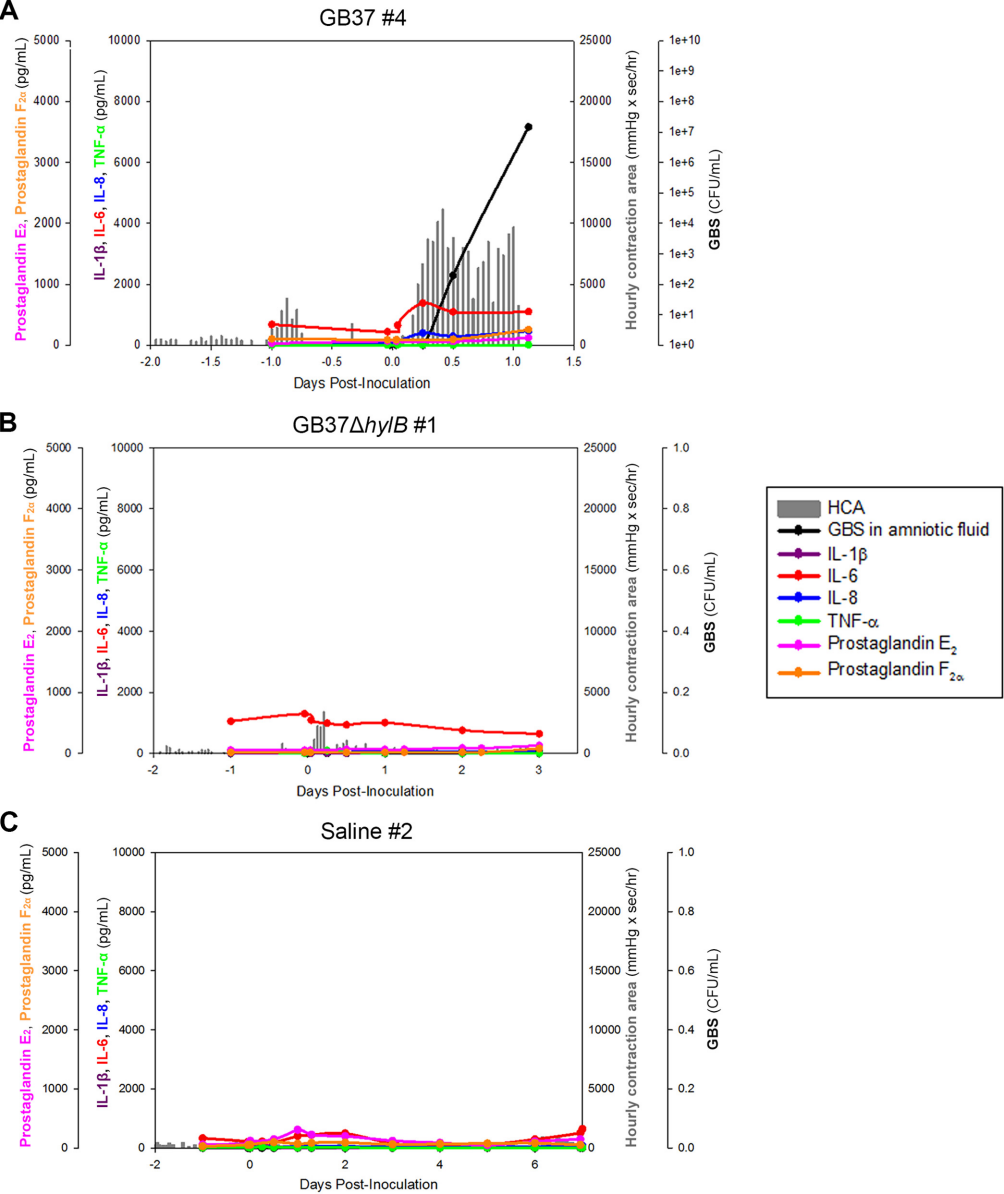
GBS hyaluronidase promotes amniotic cavity invasion and preterm labor. Chronically catheterized pregnant pigtail macaques (Macaca nemestrina) received choriodecidual inoculations of either HylB-proficient WT GBS strain GB37 (*n* = 5), an isogenic GBS strain lacking HylB (GB37Δ*hylB*, *n* = 5), or saline (*n* = 6) at 118 to 125 days gestation (term, 172 days). Uterine contractions (vertical gray lines), cytokines (IL-1β, IL-6, IL-8, and tumor necrosis factor alpha [TNF-α]), prostaglandins (PGE_2_ and PGF_2α_), and GBS CFU from the AF are shown from representative animals that received either GB37 (A), GB37Δ*hylB* (B), or saline (C).

We found that choriodecidual inoculation of the hyaluronidase-proficient GBS strain (GB37) induced preterm labor in 4/5 (80%) animals compared to 1/5 (20%) animals inoculated with the isogenic hyaluronidase-deficient strain GB37Δ*hylB* or 0/6 (0%) saline controls (Table 1 and Fig. 1). Further, all GB37-inoculated animals (5/5, 100%) experienced microbial invasion of the amniotic cavity in contrast to 1/5 animals (20%) inoculated with GB37Δ*hylB* and 0/6 (0%) saline controls (Table 1 and Fig. 1). In the GB37 group, GBS CFU was recovered from the amniotic fluid (AF) as early as 6 h postinoculation in three animals (GB37#1, GB37#2, and GB37#4), which was followed by preterm labor within 1 and 2 days (Fig. 1; see also Fig. S1 in the supplemental material). For GB37#3, GBS bacteria was recovered from the AF by 12 h after inoculation, and the AF was significantly turbid due to high bacterial burden within day 2. This was accompanied by a heavy uterine contraction pattern that resulted in cervical ripening (softening). To avoid a stillbirth due to sepsis, we proceeded with Cesarean section at 49 h after GBS inoculation for this animal. In GB37#5, infection and contractions progressed rapidly after 48 h, and the NHP was in labor by 70 h (early on day 3). In the GB37Δ*hylB* group, only one animal exhibited preterm labor and microbial invasion of the amniotic cavity (GB37Δ*hylB*#5), wherein GBS was detected in the AF at 24 h postinoculation and the animal experienced preterm labor within 70 h (Fig. 1; see also Fig. S1). The remaining four animals in this group did not exhibit microbial invasion of the amniotic cavity, preterm labor, or other adverse outcomes (Fig. 1; see also Fig. S1). Collectively, our results indicate that choriodecidual inoculation of a GBS strain with elevated hyaluronidase activity was significantly associated with adverse pregnancy outcomes compared to inoculation with the isogenic hyaluronidase-deficient GBS strain or saline controls (*P* = 0.02 GB37 versus GB37Δ*hylB*; *P* = 0.0005 GB37 versus saline). Despite the significant adverse outcomes noted in the GB37-infected group, there was no statistical difference in the peak AF cytokine levels among the three treatment groups of this study (i.e., saline, GB37, and GB37Δ*hylB*; Table 1). Interestingly, compared to our previously published studies on hyperhemolytic GBSΔ*covR*-inoculated animals (28), where we reported peak AF interleukin-6 (IL-6) and IL-8 ranging between 15 and 22 ng/ml (for animals with microbial invasion of the amniotic cavity), the GB37 group had significantly lower peak AF levels of IL-6 (*P* = 0.009) and IL-8 (*P* = 0.02). Taken together, these results suggest that increased AF cytokines such as IL-6 and IL-8 are not necessarily a predictive signature of preterm labor, particularly during infection with the immunosuppressive hyaluronidase-expressing GBS bacteria.

10.1128/mBio.03115-20.1 FIG S1 Uterine contractions, AF cytokines, prostaglandins, and bacterial CFU from choriodecidual inoculations of GB37, GB37Δ*hylB*, or saline in chronically catheterized pregnant NHPs. Download FIG S1, DOCX file, 1.5 MB. Copyright © 2021 Coleman et al. 2021 Coleman et al. This content is distributed under the terms of the Creative Commons Attribution 4.0 International license.

### GBS HylB promotes prostaglandin and matrix metalloproteinase synthesis for cervical softening.

**FIG 2:**
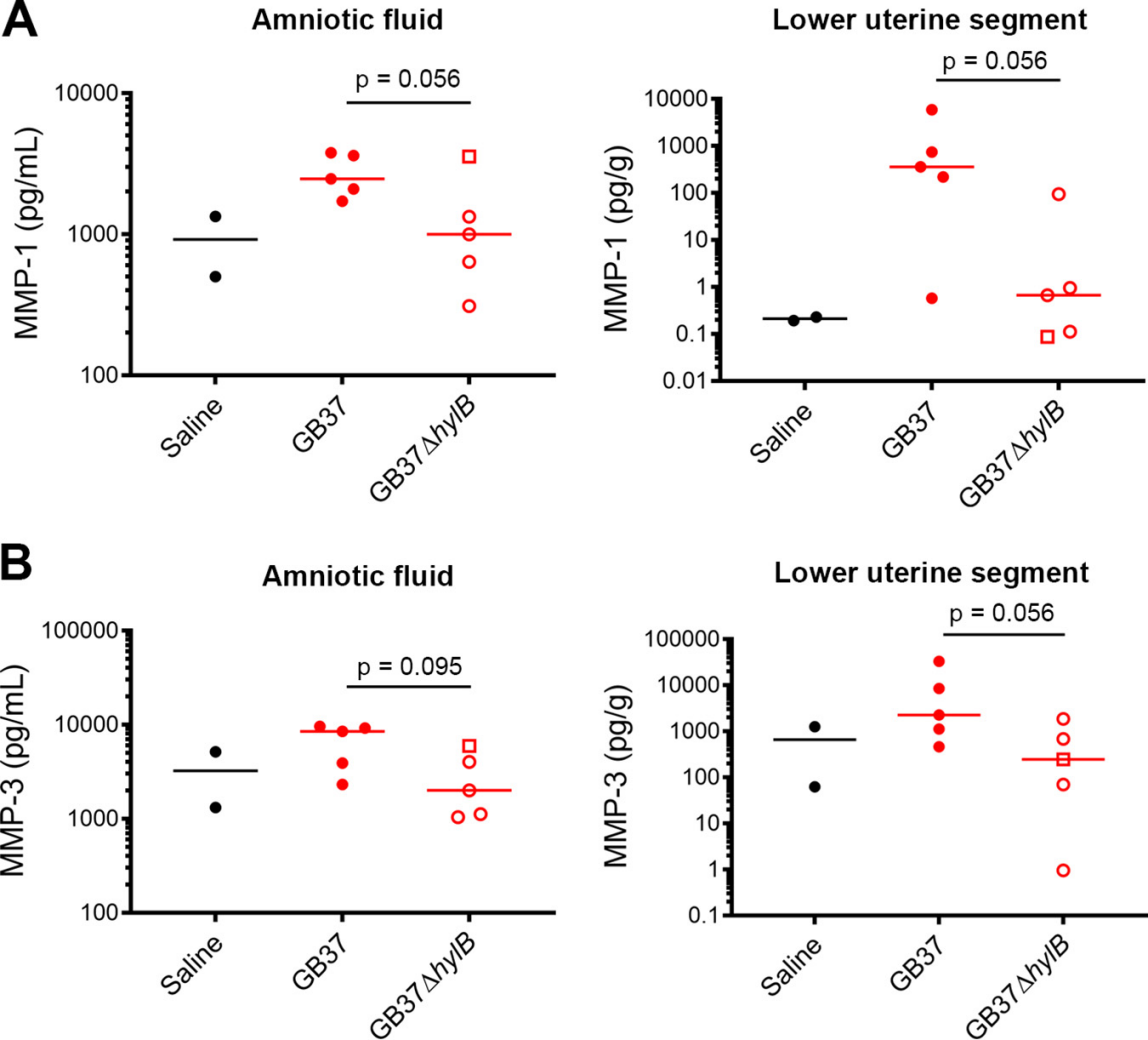
MMP-1 and MMP-3 are elevated in the amniotic fluid and lower uterus of GB37-inoculated NHP. At Cesarean section, amniotic fluid and a tissue segment from the lower uterus were collected from each animal, and each sample was analyzed for MMP-1 (A) and MMP-3 (B) levels by Luminex. Data from GB37 and GB37Δ*hylB* were compared using a Mann-Whitney test. Although *P* values between 0.055 and 0.1 are noted due to the small sample size of this nonhuman primate study, *P* values of <0.05 were considered significant. GB37Δ*hylB*#5 is designated by an open square.

Apart from increased inflammation, prostaglandin and matrix metalloproteinases (MMPs) have been implicated in inducing the thinning and softening of the cervix, leading to preterm labor (30–33). Given our observations that 80% (4/5) of the GB37-infected animals experienced preterm labor in the setting of low inflammation, we sought to determine whether infection with the hyaluronidase-proficient GBS was associated with increased prostaglandin and MMP levels, which could have contributed to cervical softening and preterm labor. We observed a relative increase in MMP-1 and MMP-3 in the AF and lower uterine segments of GB37 animals compared to GB37Δ*hylB* or saline controls (Fig. 2; see the schematic in Fig. S4a in the supplemental material for the location of the lower uterine segment). These MMPs have been implicated in preterm labor (34, 35). We also noted that peak AF prostaglandin PGF2α levels were higher in the GB37 animals compared to GB37Δ*hylB* (Table 1). Together, these data provide some potential insight into mechanisms of preterm labor due to infection by the immunosuppressive hyaluronidase-expressing GBS.

### GBS HylB promotes fetal bacteremia and fetal inflammation.

**FIG 3:**
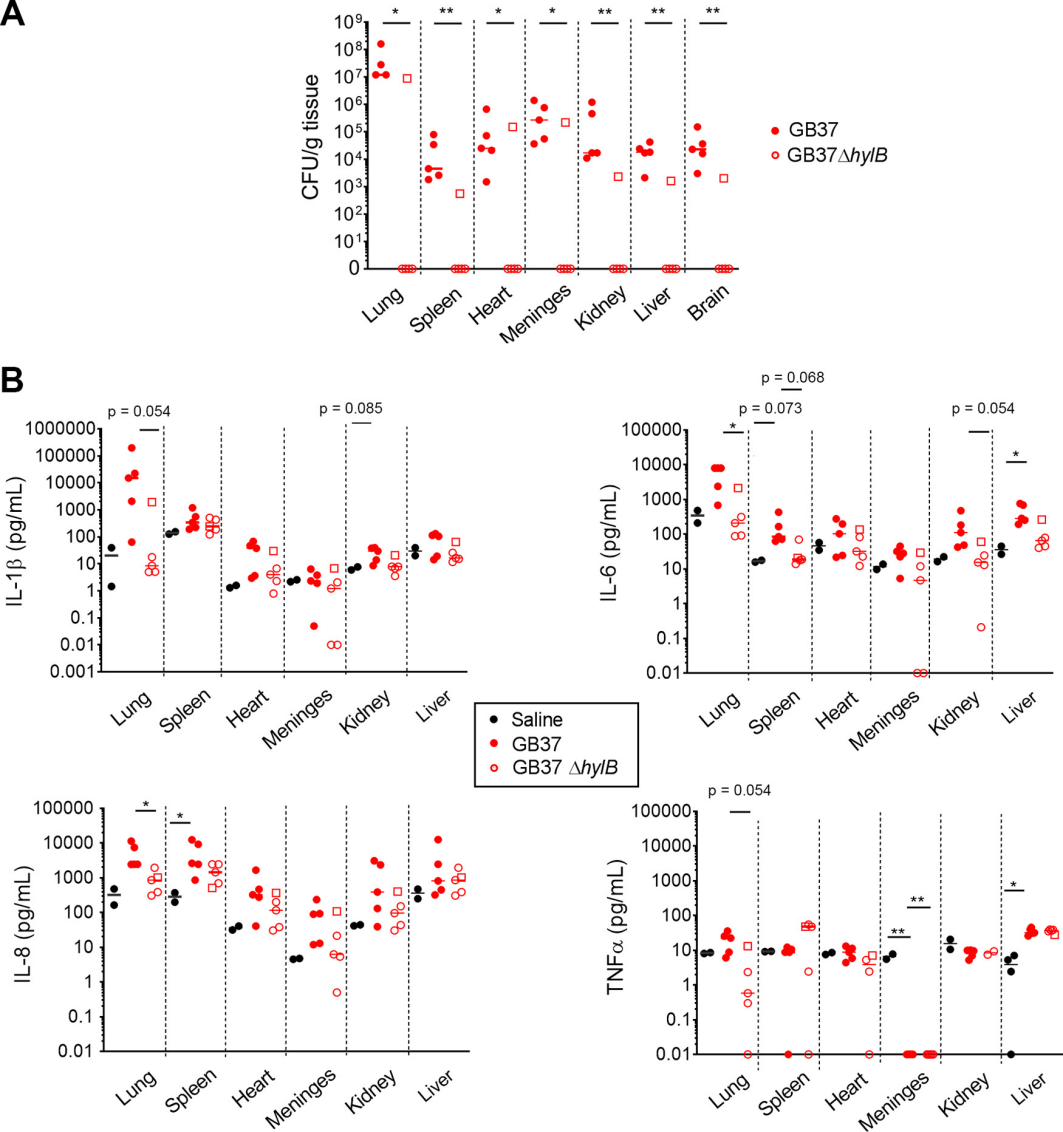
Microbial invasion of the amniotic cavity coincided with fetal bacteremia and systemic fetal inflammation. At Cesarean section, fetal tissues were harvested from chronically catheterized pregnant NHP that received a choriodecidual inoculation of saline, GB37, or GB37Δ*hylB*. GB37Δ*hylB*#5 is designated by an open square. (A) Numbers of GBS CFU obtained from various fetal tissues. Note that GBS was recovered from all fetal tissues of all animals that experienced microbial invasion of the amniotic cavity and preterm birth, including all (5/5) GB37-inoculated animals and one of five GB37Δ*hylB*-inoculated animals (GB37Δ*hylB*#5, designated by an open square). Differences in CFU between treatment groups were analyzed using the Mann-Whitney test. *, *P* < 0.05; **, *P* < 0.01. (B) Lysates from fetal tissues were examined by Luminex to evaluate differences in levels of fetal cytokines. A Kruskal-Wallis test with Dunn’s multiple-comparison test was performed. *, *P* < 0.05. *P* values between 0.05 and 0.1 are noted due to the small sample size of this nonhuman primate study but were not considered significant. Since fetal tissue lysates were not available from historical saline animals (*n* = 4) reported previously (28), only saline animals performed in the present study (*n* = 2) were included in these analyses.

In all GB37-infected animals, microbial invasion of the amniotic cavity coincided with fetal bacteremia, and GBS CFU (ranging from 10^3^ to 10^8^ CFU/g) were recovered from several fetal organs, including the lung, spleen, heart, brain, and meninges (Fig. 3A). In the GB37Δ*hylB* group, only the animal that exhibited microbial invasion of the amniotic cavity (GB37Δ*hylB*#5) exhibited fetal bacteremia. Overall, fetal bacteremia was significantly increased in animals inoculated with GB37 compared to GB37Δ*hylB* (Fig. 3A) with consistently more bacteria recovered from the fetal lung compared to the other fetal organs.

We also measured cytokines in fetal plasma and tissues from samples obtained at the study endpoint (Fig. 3B and Table 1). In contrast to low levels of proinflammatory cytokines observed in the amniotic fluid, we noted significantly increased cytokines in the fetal lung (IL-8), spleen (IL-8), and liver (IL-6) when comparing GB37-infected fetuses to saline controls (Fig. 3B). To determine the specific effect of HylB, we compared GB37 with GB37Δ*hylB* group and found significantly higher levels of IL-6 in the lung (Fig. 3B). Hematoxylin and eosin (H&E) staining of neonatal lungs supported these findings. Lungs from the GB37 group showed minimal (GB37#4) and moderate to severe (GB37#1, GB37#2, GB37#3, and GB37#5) pneumonia (see Fig. S2). In contrast, H&E staining of the GB37Δ*hylB* group showed overall minimal neutrophilic inflammation in the fetal lung (see Fig. S2). The only animal with microbial invasion of the amniotic cavity in this group, GB37Δ*hylB*#5, exhibited moderate and mixed cell-type inflammation within the lung. Myeloperoxidase (MPO; neutrophil and granulocyte marker) staining was seen in the neonatal lungs of the GB37 group with minimal staining observed in the GB37Δ*hylB* group and saline controls (see Fig. S2 and S3). Minimal to no CD68 (expressed by monocytes and macrophages) staining was observed for all three groups (see Fig. S2). Collectively, these analyses reveal that choriodecidual inoculation of hyaluronidase-expressing GBS resulted in bacterial invasion, cytokines, and inflammation in the fetal lung.

10.1128/mBio.03115-20.2 FIG S2 Histological examination of NHP fetal lung sections. (A to D) Representative H&E-stained sections from NHPs in each group are shown, including saline#3 (A), GB37#1 (B), GB37#2 (C), and GB37Δ*hylB*#2 (D). (E to H) Representative MPO-stained sections from NHPs in each group are shown, including saline#3 (E), GB37#1 (F), GB37#2 (G), and GB37Δ*hylB*#2 (H). (I to L) Representative CD68-stained sections from NHPs in each group are shown, including saline#3 (I), GB37#1 (J), GB37#1 (K), and GB37Δ*hylB*#2 (L). Note that within the GB37 group, tissues are shown reflecting harvest at 24 h (B, F, and J) and 48 h (C, G, and K). Download FIG S2, DOCX file, 0.9 MB. Copyright © 2021 Coleman et al. 2021 Coleman et al. This content is distributed under the terms of the Creative Commons Attribution 4.0 International license.

10.1128/mBio.03115-20.3 FIG S3 Quantitation of immunostaining for MPO in the fetal lung and chorioamniotic membranes. (A) The area of MPO immunostaining in the neonatal lung was significantly different between the saline and GB37 groups and between the GB37 and GB37Δ*hylB* groups. GB37Δ*hylB*#5 is designated by an open square. A one-way ANOVA with Tukey’s posttest was used to compare groups. (B) The area of MPO immunostaining in the chorioamniotic membranes was not significantly different among the groups in the amnion, chorion, or decidua. GB37Δ*hylB*#5 is designated by an open square. A one-way ANOVA with Tukey’s posttest was used to compare groups. Download FIG S3, DOCX file, 0.1 MB. Copyright © 2021 Coleman et al. 2021 Coleman et al. This content is distributed under the terms of the Creative Commons Attribution 4.0 International license.

10.1128/mBio.03115-20.4 FIG S4 GB37-inoculated NHPs experienced enhanced infiltration of CD8^+^ T cells and phagocytes to the maternal fetal interface. At Cesarean section, biopsy specimens from the uterus and placenta, as well as maternal and fetal blood, were obtained. (A) A schematic depicting the biopsy sites is shown. (B) Samples were processed into single-cell suspensions, stained, and analyzed by flow cytometry for various immune cell markers (indicated in the figure). GB37Δ*hylB* #5 is designated by an open square. A Welch’s test was used to evaluate differences in immune cell populations between GB37- and GB37Δ*hylB*-treated animals at each site. Data from two saline controls performed as a part of the present study are included, but similar analyses were not previously performed with historical saline controls (*n* = 4). Download FIG S4, DOCX file, 0.4 MB. Copyright © 2021 Coleman et al. 2021 Coleman et al. This content is distributed under the terms of the Creative Commons Attribution 4.0 International license.

### GBS HylB induced greater infiltration of phagocytes to the maternal-fetal interface.

**FIG 4:**
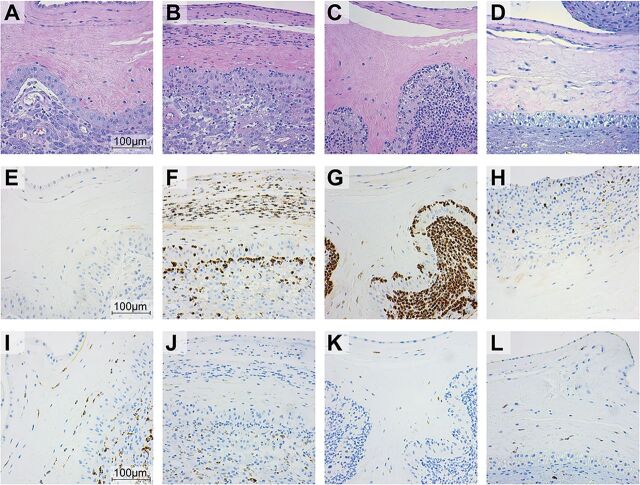
Histological examination of the placental membranes revealed increased neutrophil infiltration in GB37-infected animals. (A to D) H&E staining of NHP placental sections. Representative H&E-stained sections from NHPs in each group are shown, including saline#3 (A), GB37#1 (B), GB37#2 (C), and GB37Δ*hylB*#2 (D). (E to H) Representative MPO-stained sections from NHPs in each group, including saline#3 (E), GB37#1 (F), GB37#2 (G), and GB37Δ*hylB*#2 (H). (I to L) Representative CD68-stained sections from NHPs in each group, including saline#2 (I), GB37#1 (J), GB37#2 (K), and GB37Δ*hylB*#2 (L). For the GB37 group, tissues in panels B, F, and J were taken 24 h after inoculation and tissues in panels C, G, and K were harvested 48 h after inoculation.

We examined the chorioamniotic membranes at the inoculation site using H&E, MPO, and CD68 to assess inflammation and tissue injury at the maternal-fetal interface. As expected, saline controls revealed minimal to no neutrophil infiltration (Fig. 4A; see also Fig. S3), whereas chorioamniotic membranes from animals in the GB37 group exhibited moderate to severe neutrophilic inflammation (Fig. 4B and C; see also Fig. S3). Conversely, placental membranes from the GB37Δ*hylB* group had minimal infiltration (Fig. 4D; see also Fig. S3), except for GB37Δ*hylB*#5, which exhibited moderate neutrophilic inflammation (data not shown). In contrast, CD68 staining (a marker for monocytes and macrophages) was similar across all three groups (saline, GB37, and GB37Δ*hylB*; Fig. 4I to L), indicating that neutrophils rather than macrophages are predominantly recruited to the choriodecidual interface during GBS infections. Notably, we also observed increased neutrophil levels in the chorioamniotic membranes and miduterine muscle by flow cytometry in GB37-treated animals compared to those inoculated with the Δ*hylB* strain (see Fig. S4). Collectively, these data indicate that immune cells typically involved in the host response to tissue damage and/or pathogen control are increased in the reproductive tissues of animals inoculated with GB37 versus GB37Δ*hylB* or saline controls.

### Digital spatial profiling of the placenta revealed minimal immune signatures to GBS hyaluronidase.

**FIG 5:**
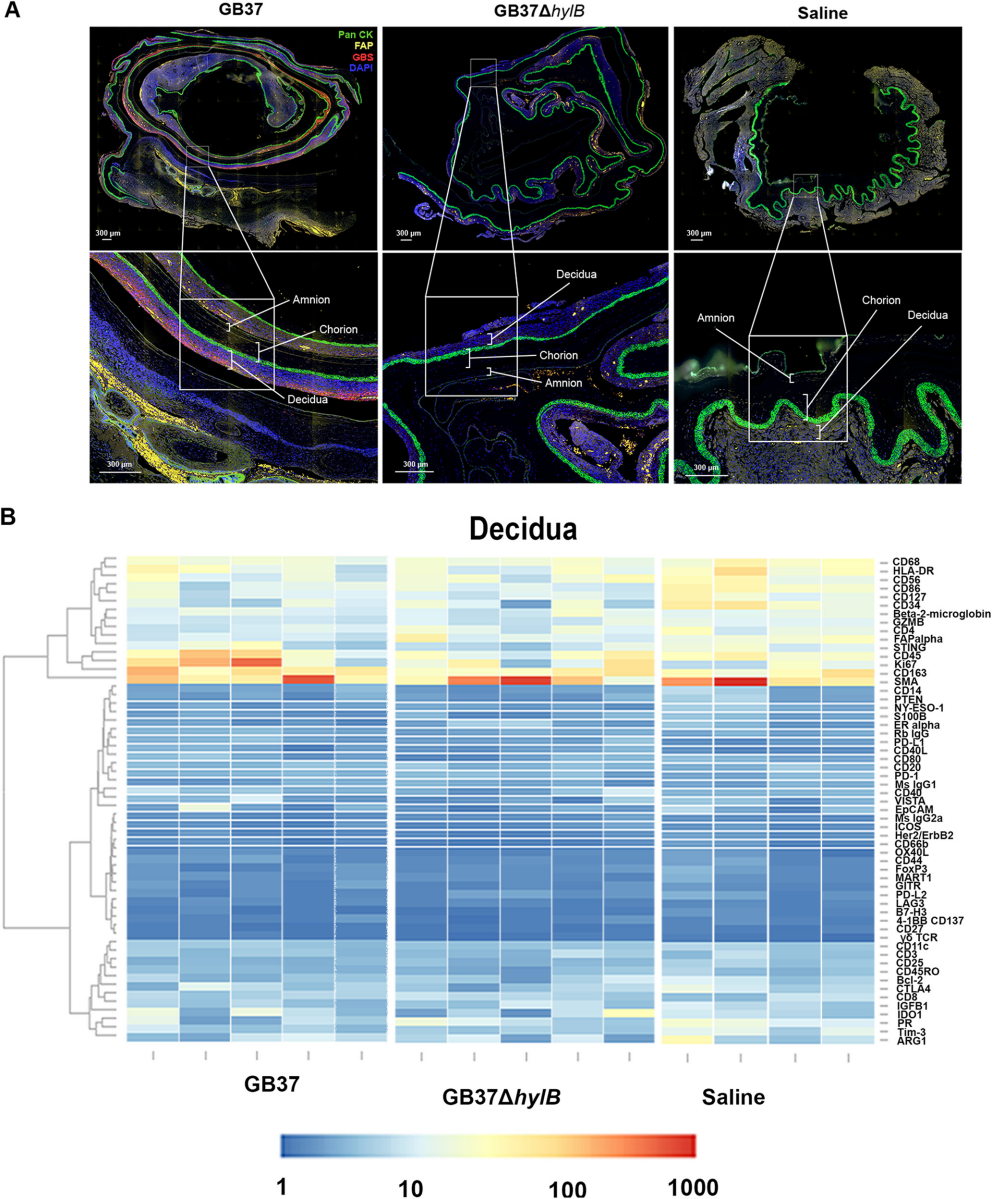

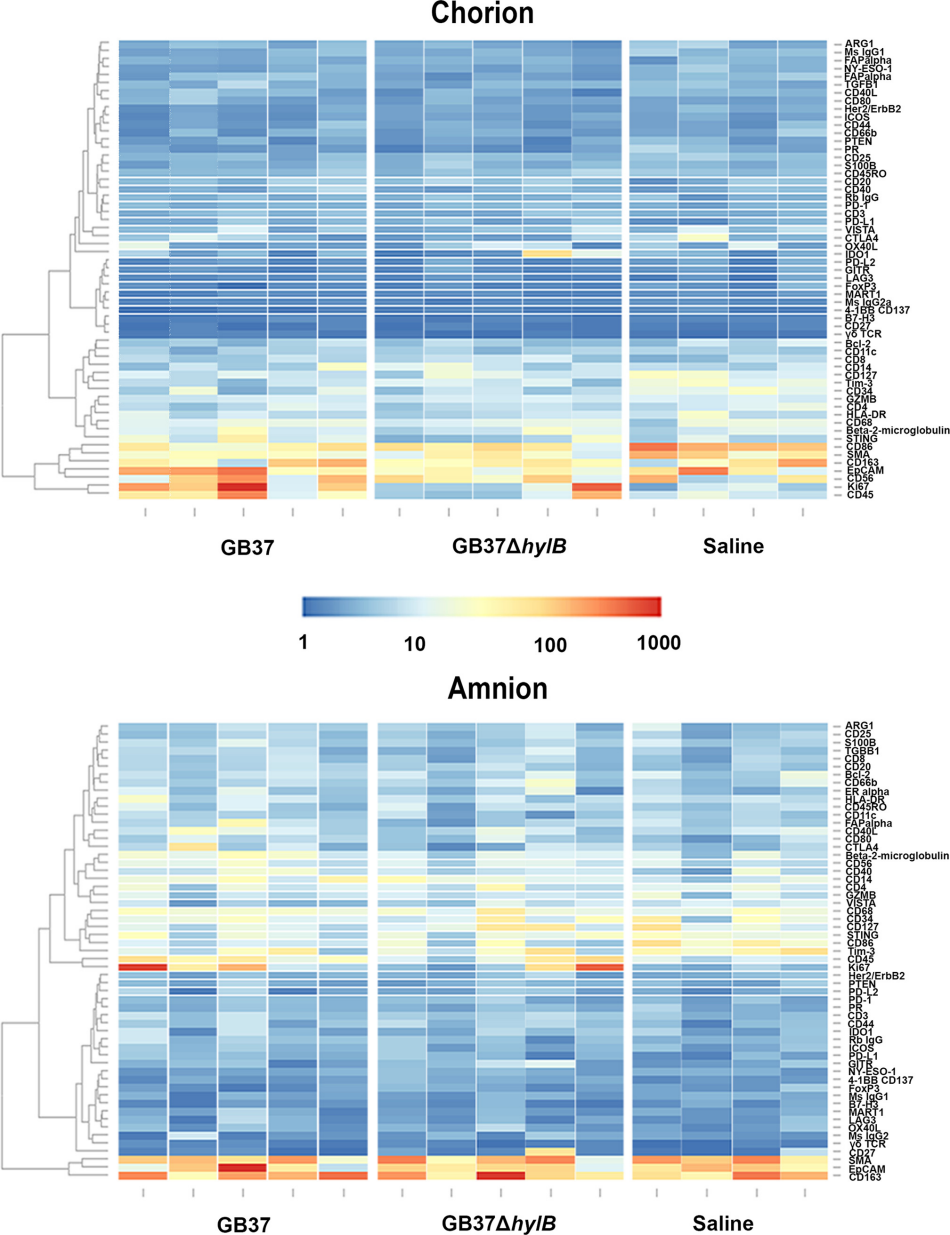
Digital spatial profiling of placental tissues revealed few differential expressions of immune signatures GBS hyaluronidase. (A) Representative placental sections from NHP in each treatment group (GB37#2, GB37Δ*hylB*#2, and saline#3). We treated each placental section with fluorescent anti-pan cytokeratin (Pan CK, green), anti-fibronectin attachment protein (FAP, yellow), anti-GBS (red), and DAPI (blue) and then identified the decidua, chorion, and amnion within each section as distinct ROIs. Each discrete ROI (i.e., chorion, amnion, and decidua) was analyzed for analyte abundance. (B) Heatmaps showing analyte abundance (normalized by the signal/noise ratio) in the decidua, chorion, and amnion of each animal in the GB37 (*n* = 5), GB37Δ*hylB* (*n* = 5), and saline (*n* = 4) groups are shown.

To obtain greater spatial resolution of the cellular events and signaling cascades occurring at the uterine mucosa, we analyzed paraffin-embedded placental membranes using the GeoMx digital spatial profiling (DSP) platform (NanoString Technologies) (Fig. 5). The DSP scanned whole tissue image shown in Fig. 5A is obtained by stitching together images that are obtained from single Field Of View (FOV): each FOV image has a dimension of 2048 pixel × 2048 pixel, which equals 665.6 μm × 665.6 μm. Regions of interest (ROIs) comprising the decidua, chorion, and amnion in each animal were selected with preferential targeting of areas with GBS (Fig. 5A), and 62 antibody probes coupled to cleavable oligonucleotide tags were hybridized and quantified. Counts were mapped to tissue location to yield a spatially resolved profile of analyte abundance (Fig. 5B). Intriguingly, while GBS was detected in the decidua of all GB37-treated animals (Fig. 5A) and in only one GB37Δ*hylB* animal, no significant differences in inflammation-associated analytes (e.g., CD45, CD68, STING, CD86, and CD56) were observed between the GBS groups for any tissue type (amnion, chorion, decidua) at these ROIs (Fig. 5B; see also Fig. S5). Conversely, some markers associated with inflammation were significantly lower in GB37 animals compared to saline controls, including the macrophage marker CD14 in the decidua and amnion and CD86 (T cell costimulatory molecule on APCs) in the decidua, chorion, and amnion (Fig. 5B; see also Fig. S6). These data indicate that even in tissues sites where GB37 were detected by fluorescence microscopy (Fig. 5A), some inflammatory markers were dampened relative to baseline. Together, digital spatial profiling showed that choriodecidual inoculation with GB37 did not induce a remarkable proinflammatory signature in the amnion, chorion, or decidua.

10.1128/mBio.03115-20.5 FIG S5 Digital spatial profiling analyte fold change: GB37 versus GB37Δ*hylB*. Analyte abundance in distinct placental regions from GB37Δ*hylB*-inoculated NHPs and saline-treated NHPs were obtained by digital spatial profiling (Nanostring Technologies). Fold changes in analyte abundance (GB37Δ*hylB* over GB37) were log_2_ transformed and analyzed by a linear mixed model in R version 3.6.2. Significance tests were controlled for false discovery rate. Download FIG S5, DOCX file, 0.3 MB. Copyright © 2021 Coleman et al. 2021 Coleman et al. This content is distributed under the terms of the Creative Commons Attribution 4.0 International license.

10.1128/mBio.03115-20.6 FIG S6 Digital spatial profiling analyte fold change: GB37 versus saline. Analyte abundance in distinct placental regions from GB37-inoculated NHPs and saline-treated NHPs were obtained by digital spatial profiling (Nanostring Technologies). Fold changes in analyte abundance (GB37 over saline) were log_2_ transformed and analyzed by a linear mixed model in R version 3.6.2. Significance tests were controlled for false discovery rate. White asterisk, *P* < 0.05. Download FIG S6, DOCX file, 0.3 MB. Copyright © 2021 Coleman et al. 2021 Coleman et al. This content is distributed under the terms of the Creative Commons Attribution 4.0 International license.

### HylB promotes resistance to antimicrobial effects of neutrophils.

**FIG 6:**
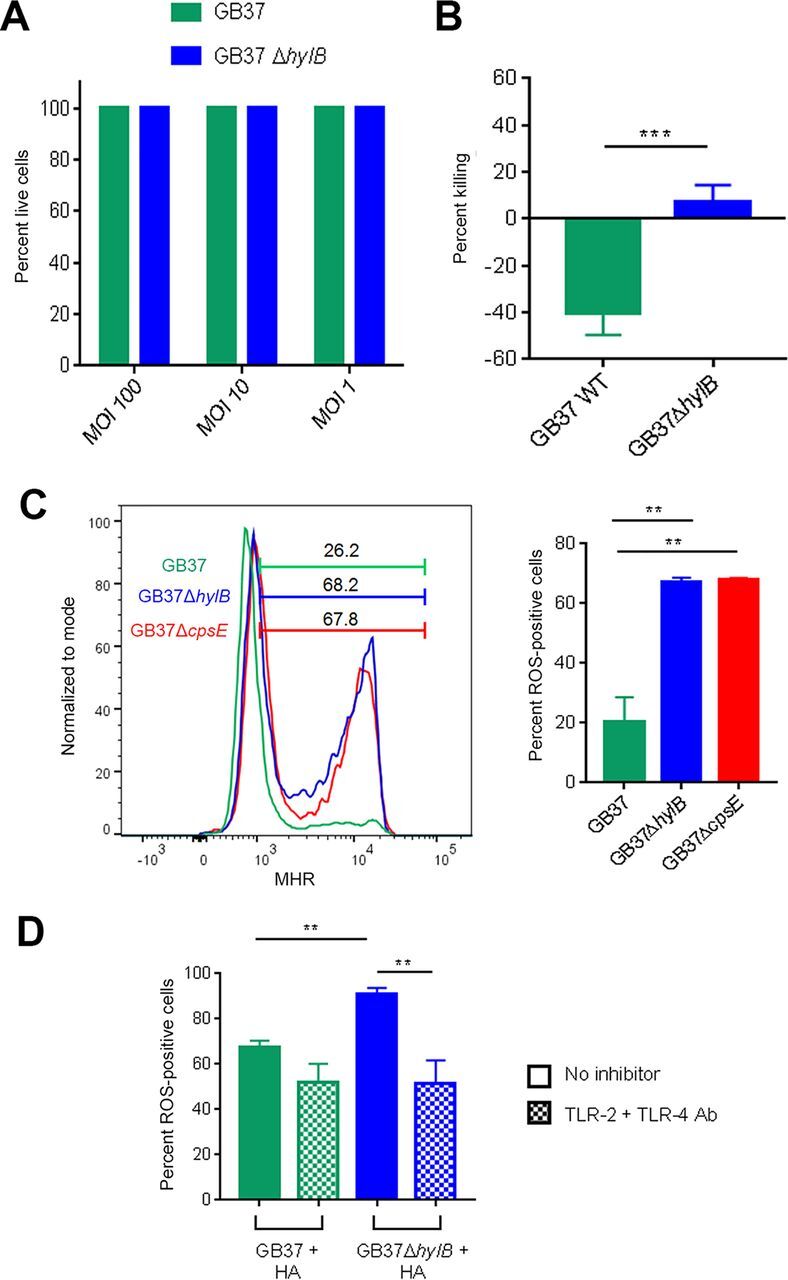
GBS HylB evades neutrophil killing independently of cell death by interfering with TLR-2/4 signaling. (A) Primary human neutrophils were isolated from fresh adult blood, exposed to GB37 or GB37Δ*hylB* at MOIs 100, 10, or 1 for 4 h, and then examined for cell death by LDH release. The percent live cells was calculated relative to Triton X-100-treated positive controls (0% live cells) and PBS-treated negative controls (100% live cells). (B) GB37 or GB37Δ*hylB* was exposed to primary human neutrophils isolated from fresh blood (MOI of 1) for 1 h. The percent killing was calculated as the number of CFU recovered after incubation with neutrophils out of the number of the number CFU recovered after incubation without neutrophils × 100. Differences among groups were determined by a paired *t* test. (C) Primary human neutrophils were isolated as described above, pretreated with dihydrorhodamine-123 (DHR), and then exposed to GB37 or GB37Δ*hylB* (MOI of 100). Since GBS capsule can suppress neutrophil ROS generation by blocking Siglec 9 (62), the GB37Δ*cpsE* was included as a control. The conversion of DHR to fluorescent MHR indicates ROS production in cells and was measured by flow cytometry at 60 min postinfection. Differences among treatment groups were determined by one-way analysis of variance (ANOVA). (D) Filtered supernatants of stationary-phase GB37 or GB37Δ*hylB* liquid cultures were incubated with HA for 18 h to allow for enzymatic digestion of HA. Meanwhile, primary human neutrophils were pretreated with 10 μg/ml anti-TLR-2 antibody (Invivogen) plus 10 μg/ml anti-TLR-4 antibody (Invivogen) or vehicle control. Neutrophils were treated with DHR and then exposed to the digested HA solutions from each strain for 60 min. As described above, ROS production in cells was measured by detecting fluorescent MHR via flow cytometry. Differences in MHR-positive cells among treatment groups were determined by one-way ANOVA. *, *P* < 0.05; **, *P* < 0.01; ***, *P* < 0.001; ****, *P* < 0.0001; ns, *P* ≥ 0.05.

Given our observations of neutrophil recruitment in the chorioamniotic membranes and uterus of GB37-infected animals (Fig. 4; see also Fig. S4), with minimal proinflammatory events in the placenta (Fig. 5B), we sought to determine whether GBS hyaluronidase plays a role in dampening neutrophil defenses, which are critical in protecting the host against GBS infection (28, 36). We previously observed that hyperhemolytic strains of GBS circumvented the neutrophil response, in part, by inducing neutrophil cell death (28). Here, no significant cell death was observed when fresh adult human neutrophils were treated with GB37 or GB37Δ*hylB* (Fig. 6A). Given that HA is present on the cell surface of neutrophils (37), we next sought to determine whether HylB might enable GBS to resist the microbicidal activity of neutrophils. We found neutrophils were less able to kill GB37 compared to GB37Δ*hylB* (Fig. 6B; see also Fig. S8a), indicating that hyaluronidase enables GBS to subvert this critical host defense.

10.1128/mBio.03115-20.7 FIG S7 Digital spatial profiling analyte fold change: GB37Δ*hylB* versus saline. Analyte abundance in distinct placental regions from GB37Δ*hylB*-inoculated NHPs and saline-treated NHPs were obtained by digital spatial profiling (Nanostring Technologies). Fold changes in analyte abundance (GB37Δ*hylB* over saline) were log_2_ transformed and analyzed by a linear mixed model in R version 3.6.2. Significance tests were controlled for false discovery rate. White asterisk, *P* < 0.05. Download FIG S7, DOCX file, 0.3 MB. Copyright © 2021 Coleman et al. 2021 Coleman et al. This content is distributed under the terms of the Creative Commons Attribution 4.0 International license.

10.1128/mBio.03115-20.8 FIG S8 Neutrophils were isolated from human maternal blood and umbilical cord (i.e., fetal) blood. (A) GB37 or GB37Δ*hylB* were exposed to maternal or fetal neutrophils (MOI of 1) for one hour. The percent killing was calculated as the number of CFU recovered after incubation with neutrophils out of the number of the number CFU recovered after incubation without neutrophils × 100. Differences among groups were determined using a paired *t* test. *, *P* < 0.05. (B) Maternal or fetal neutrophils were pretreated with dihydrorhodamine-123 (DHR) and then exposed to GB37 (green) or GB37Δ*hylB* (blue) (MOI of 100). The conversion of DHR to fluorescent MHR indicates ROS production in cells and was measured by flow cytometry at 60 min postinfection. Download FIG S8, DOCX file, 0.1 MB. Copyright © 2021 Coleman et al. 2021 Coleman et al. This content is distributed under the terms of the Creative Commons Attribution 4.0 International license.

To understand the mechanisms by which HylB may facilitate GBS resistance to neutrophil killing, we measured the production of ROS in neutrophils in the presence of hyaluronidase-proficient or deficient GBS using a flow cytometry-based assay (see the supplemental methods [Text S1]). We noted decreased production of ROS in neutrophils exposed to GB37 compared to GB37Δ*hylB*, demonstrating that HylB may have blunted the ability of neutrophils to generate ROS (Fig. 6C). This effect was also observed with maternal and fetal neutrophils (see Fig. S8b). Next, we sought to determine whether HylB-mediated TLR-2/4 interference could explain the differences in neutrophil ROS production elicited by the wild-type (WT) and Δ*hylB* strains. To test this, supernatants from GB37 or GB37Δ*hylB* were incubated with HA to allow for enzymatic digestion, as described previously (21). Then, HA digests from each strain were exposed to human neutrophils pretreated with anti-TLR-2 and anti-TLR-4 antibodies or vehicle alone, and ROS production was measured. As expected, GB37-HA digests induced less ROS production than GB37Δ*hylB*-HA digests. Intriguingly, when neutrophils were pretreated with both TLR inhibitors, this effect was lost (Fig. 6D). These findings suggest that proinflammatory HA fragments from hyaluronidase-deficient GBS promote ROS production in neutrophils by engaging TLR-2/4 signaling. Furthermore, these data indicate that GBS may avoid neutrophil killing by using HylB to cleave HA into fragments that blunt TLR2/4 signaling and subsequently diminish ROS production. Taken together, our findings are the first to demonstrate that HylB promotes GBS resistance to neutrophils, which contributes to adverse pregnancy outcomes in pregnant nonhuman primates.

10.1128/mBio.03115-20.10 TEXT S1 Details regarding the study design, generation of GB37Δ*cpsE* mutant, catheterization of pregnant NHP, sample collection from NHP, analysis of cytokines, MMP, flow cytometry, immunostaining, and isolation of neutrophils from human blood, neutrophil assays, and statistical analyses. Download Text S1, DOCX file, 0.04 MB. Copyright © 2021 Coleman et al. 2021 Coleman et al. This content is distributed under the terms of the Creative Commons Attribution 4.0 International license.

## DISCUSSION

Understanding mechanisms that GBS use to subvert host defenses in its transition from an asymptomatic colonizer to invasive pathogen is essential for the development of improved treatment and prevention strategies for adverse perinatal outcomes associated with GBS. We previously showed that hyaluronidase (HylB) activity was greater in GBS strains isolated from cases of preterm birth and neonatal infection compared to commensal isolates (22) and that HylB facilitated ascending GBS infection in mice (22). Our data in the chronically catheterized NHP model indicate that HylB is a crucial virulence factor that enables GBS to rapidly invade the amniotic cavity and induce preterm labor. Overall, while we noted a relative suppression of inflammation in key placental, uterine, and amniotic fluid samples, preterm labor was induced by HylB-expressing GBS.

Despite clear phenotypic differences in pregnancy outcomes in GB37-treated animals compared to the GB37Δ*hylB* group, differences in peak AF cytokines and immune signaling in the placenta were not as striking. These results are also in stark contrast to a pregnant NHP model using a hyperhemolytic/hyperpigmented GBS strain wherein we observed significantly higher levels of IL-6 and IL-8 in the AF and upregulation of proinflammatory genes in the placenta compared to NHPs inoculated with a nonhemolytic strain and saline controls (28). In contrast, results from this study indicate that HylB may suppress inflammatory responses at the maternal-fetal interface to promote bacterial replication and dissemination. Luminex (Fig. 1), digital spatial profiling (Fig. 5), and *in vitro* studies (Fig. 6), indicate immune suppression, which can be due in part to inhibition of TLR-2/4 signaling by HylB-expressing GBS (Fig. 6D). These data are consistent with observations that term placenta expresses high levels of TLR-2 and TLR-4, with stronger expression of TLR-2 in immune cells and TLR-4 in syncytiotrophoblasts and fibroblasts (38). Expression of TLR-4 was also observed on the apical side of amniotic epithelium (39, 40), suggesting immune suppression of placenta could also occur after microbial invasion of the amniotic cavity. Together, these observations show that immune cells (maternal/fetal), trophoblasts, and other cells within the placenta are susceptible to immune suppression by the GBS hyaluronidase.

Although we observed relative suppression of inflammation at the maternal-fetal interface, fetal cytokines were induced in the setting of fetal bacteremia (Fig. 3). Given the *in vivo* and *in vitro* data obtained in this study, we propose that HylB may dampen proinflammatory cascades and microbicidal activity of first-line innate immune defenses such as neutrophils, thereby promoting bacterial replication and invasion of the amniotic cavity and fetus. We hypothesize that once bacteria have invaded the fetal niche, other proinflammatory, surface-associated bacterial factors (e.g., capsular polysaccharides [41], surface immunogenic protein [42], pilus [43]) overcome the immune-dampening effects of GBS HylB-digested HA fragments, triggering host PRRs and resulting in systemic fetal inflammation. A systemic fetal inflammatory response in GB37-infected fetuses may have triggered parturition in these animals, akin to fetal inflammatory response syndrome, which has been linked to preterm labor in humans (44, 45). Premature cervical softening in associated with increased MMP levels (Fig. 3) and prostaglandins, may have also played a role in early labor onset (46–48). Further research on the effect of HylB on host pathways during GBS infection is critical to understanding the multifaceted etiologies of GBS-associated preterm labor.

Limitations of our study include the lack of information on cellular events occurring in the amniotic fluid due to low cell recovery. In addition, we were unable to observe the cellular events at the placenta and uterus immediately after bacterial inoculation prior to preterm labor and delivery. Accordingly, it is possible that we did not capture events that may lend greater insight into the kinetics of GBS HylB-mediated immune suppression. For instance, as GB37Δ*hylB* lacks the ability to degrade HA into anti-inflammatory dimers (21), GB37Δ*hylB* inoculation may have induced rapid recruitment of neutrophils via controlled inflammation, resulting in swift clearance of this strain before microbial invasion of the amniotic cavity could occur (in all animals except GB37Δ*hylB*#5). This idea is supported by our previous findings in an intraperitoneal murine model of infection, where we observed greater neutrophil recruitment to the peritoneal fluid of GB37Δ*hylB*-inoculated mice within 2 h postinfection and less bacterial dissemination by 48 compared to GB37-inoculated mice (29). In the present study, bacterial clearance and resolution of inflammation within the chorioamniotic membranes in four of five GB37Δ*hylB-*treated NHPs could explain the lower neutrophil frequencies in the membranes and uterine tissues observed 3 days after inoculation. A time-controlled experiment in the NHP pregnancy model would lend greater insight into the differences in neutrophil recruitment and inflammatory events immediately following choriodecidual infection with GB37 or GB37Δ*hylB*.

Together, our findings establish HylB as a potent virulence factor that allows GBS to circumvent neutrophil responses, invade the amniotic cavity, and cause fetal bacteremia and preterm labor in an animal model that closely resembles human pregnancy. The chronically catheterized model enabled us to evaluate uterine contraction patterns, cervical dilation, microbial invasion of the amniotic cavity, and amniotic fluid responses in real time, which is not possible in the pregnant mouse model. Data obtained from the NHP model show that the hyaluronidase-proficient WT GB37 strain induced significant uterine contraction leading to cervical dilation (similar to human preterm labor) as early as 6 h postinoculation and also significantly delayed and diminished placental, amniotic, uterine, and myometrial immune responses as seen via Luminex, flow cytometry, and digital spatial profiling. Accordingly, these results point to HylB as a potential therapeutic target for invasive GBS disease during pregnancy. Several known hyaluronidase inhibitors exist and have been proposed as therapeutics for other HA-mediated pathology, including sexually transmitted infections (49, 50), venom wounds (51, 52), and cancer (53–55). Identification of a HylB-specific inhibitor could lead to new treatments for GBS and have far-reaching impacts on maternal and neonatal health worldwide.

## MATERIALS AND METHODS

### Ethics statement.

Written informed patient consent for donation of adult human blood was obtained with approval from the Seattle Children’s Research Institute Institutional Review Board (protocol 11117). Children under the age of 18 years were not recruited for donation of adult human blood for protocol 11117. Written informed patient consent for donation of maternal and cord blood was obtained from the University of Washington Institutional Review Board (protocol 34004). These studies were performed per the Principles in the WMA Declaration of Helsinki and Department of Health and Human Services Belmont Report.

All animal experiments were carried out in strict accordance with the recommendations in the *Guide for the Care and Use of Laboratory Animals* of the National Research Council and the Weatherall report, “The use of nonhuman primates in research.” The protocol was approved by the University of Washington Institutional Animal Care and Use Committee (permit 4165-01). All surgery was performed under general anesthesia, and all efforts were made to minimize pain and distress.

### Chemicals.

Unless indicated otherwise, all chemicals were purchased from Sigma-Aldrich.

### Bacterial strains.

GBS strains used in the NHP model were derived from strain GB37 (serotype V, multilocus sequence type 1), which was isolated from the blood of a septic human neonate with early onset disease (56). This strain is neither hemolytic nor pigmented (57) but exhibits increased hyaluronidase activity compared to other GBS clinical isolates (29). The isogenic strain GB37Δ*hylB* lacks hyaluronidase and was described previously (29). The GB37Δ*cpsE* strain was derived from WT GB37 using methods described previously (22, 58) (see Text S1 for details). Cultures of GBS were grown in tryptic soy broth (TSB) or tryptic soy agar (TSA; Difco Laboratories) at 37°C with 5% CO_2_. For inoculations in the NHP model, GBS strains were grown to mid-log phase (OD_600_ = 0.27), and 1 × 10^8^ CFU in 1 ml of sterile PBS was inoculated into the choriodecidual space, as described below and previously (27, 28). Similarly, for *in vitro* studies, GBS strains were grown to mid-log phase (OD_600_ = 0.27), washed, and resuspended in sterile phosphate-buffered saline (PBS) prior to infection unless otherwise noted.

### Chronically catheterized NHP model.

As described previously (28), pregnant pigtail macaques (Macaca nemestrina) were time-mated, and fetal age was determined using early ultrasound. Animals were provided drinking water at all times and fed commercial monkey chow, supplemented daily with fruits and vegetables. The temperature in the animal quarters was maintained between 72 and 82°F. Each animal was conditioned to a nylon jacket/tether system for several weeks before surgery, which allowed for free movement within the cage while protecting the catheters. Between days 116 and 125 of pregnancy (term = 172 days), NHPs were catheterized by laparotomic surgical implantation into the maternal femoral vein, amniotic cavity, and choriodecidual interface in the lower uterine segment (i.e., between the uterine muscle and fetal membranes, external to amniotic cavity). For detailed methods of catheterization surgery, see Text S1 in the supplemental material.

The experiment was initiated when a catheterized pregnant NHP received one of two experimental treatments: choriodecidual inoculation of either GBS strain GB37 (*n* = 5) or the isogenic strain GB37Δ*hylB* (*n* = 5). Two chronically catheterized NHP that were inoculated with sterile saline were also included, along with four saline controls performed in previous studies (27, 59).

AF and maternal blood were collected for culture, cytokine, and prostaglandin analysis during each experiment. Intra-amniotic pressure was continuously recorded, digitized, and analyzed as described previously (27, 28). The integrated area under the curve for intrauterine pressure was used as a measure of uterine activity and reported as the hourly contraction area (HCA; mm Hg ⋅ s/h) over 24 h. Preterm labor was defined as progressive cervical dilation associated with increased and sustained uterine activity (>10,000 mm Hg ⋅ s/h). Cesarean section was performed at the following experimental endpoints to allow for tissue collection: (i) preterm labor, (ii) 3 days after GBS inoculation if preterm labor did not occur, or (iii) 7 days after saline inoculation (27, 28). After Cesarean section, fetuses were euthanized by barbiturate overdose, followed by exsanguination and fetal necropsy (27, 28). Complete gross and histopathologic examinations were performed. For details on sample collection, see Text S1 in the supplemental material.

### Placental and fetal lung histology.

A board-certified veterinary pathologist (A.B.) performed blinded histopathologic examination of the fetal and placental tissues. H&E-stained, full-thickness paraffin sections (placental disc, umbilical cord, and fetal membrane roll) were examined from each case to exclude inflammation, necrosis, fetal vascular thrombosis, or other histopathological findings. Chorioamnionitis was diagnosed by the presence of a neutrophilic infiltrate at the chorion-decidua junction (mild) or amniochorion junction (moderate or severe). For histologic examination of the fetal lung, two to three randomly selected fixed fetal lung tissues were embedded in paraffin, and sections were stained with H&E. We also performed immunostaining for MPO (a granulocyte marker) and CD68 (a macrophage marker) on the chorioamniotic membranes and fetal lung tissues (see Text S1 in the supplemental material).

### Digital spatial profiling.

GeoMx digital spatial profiling (DSP) was performed at NanoString Technologies in Seattle, WA. Formalin-fixed, paraffin-embedded placental sections from animals from each group were incubated with fluorescent probes and a multiplex cocktail of primary antibodies with photocleavable oligonucleotides (i.e., a validated DSP human-immune oncology protein panel; NanoString Technologies). The fluorescent markers included anti-pan cytokeratin-Alexa Fluor 488 (Pan-CK, clone AE1/AE3; Novusbio), anti-fibroblast activation protein-Alexa-Fluor 594 (FAP, clone SP325; Abcam), SYTO 83 for nuclei visualization (Thermo Fisher), and anti-GBS-Alexa Fluor 647 (clone ab53584; Abcam). Sections were magnified to 20×, and ROIs comprising the decidua, chorion, and amnion from each animal were selected based on tissue morphology (Fig. 5B). Each region of interest was then exposed to UV illumination with a double digital mirror device molecule, which cleaved the DNA oligonucleotides into the aqueous layer above the tissue slice. The oligonucleotides in the eluent were collected via microcapillary aspiration and transferred to an individual well of a microtiter plate. Oligonucleotides were then hybridized to nCounter optical barcodes (NanoString Technologies) to permit *ex situ* digital counting of each analyte. Briefly, hybridization of oligonucleotides to optical barcodes were performed at 65°C in a thermocycler. After hybridization, samples were processed using the nCounter prep station and digital analyzer. Data were normalized to technical controls and area. To generate signal/noise ratios, data were calculated relative to isotype controls.

### Neutrophil assays.

For detailed methods on neutrophil isolation from whole human blood, see the supplemental material. To measure neutrophil death, neutrophils (1 × 10^5^ cells in RPMI containing L-glutamine) were exposed to GB37 or GB37Δ*hylB* (MOI of 100, 10, or 1) for 4 h at 37°C, and the release of LDH (lactate dehydrogenase) into cell supernatants was quantified using the LDH assay kit (TaKaRa) according to the manufacturer’s instructions. The percent cell death was calculated relative to 0.1% Triton X-100-treated (100% cell death) and PBS-treated (0% cell death) controls. To measure neutrophil killing of GBS, neutrophils (1 × 10^6^) were incubated with GB37 or GB37Δ*hylB* at an MOI of 1 in RPMI per g for 1 h at 37°C, as described previously (28, 60). Triton X-100 (0.1%) was added to lyse neutrophils and release intracellular bacteria, and total bacteria (intracellular and extracellular) were enumerated by serial dilution plating on TSA. The percent killing was calculated as the number of CFU recovered in the presence of neutrophils over the number of CFU recovered in the absence of neutrophils × 100. To measure ROS production, neutrophils (1 × 10^6^ cells/ml in RPMI per g) were preincubated with 84 μM dihydrorhodamine-123 (DHR; in 0.28% dimethyl sulfoxide) at 37°C for 20 min, as described previously (28), and then exposed to GB37, GB37Δ*hylB*, or GB37Δ*cpsE* (MOI of 100) for 60 min. The fluorescence intensity of cells (which measures DHR oxidation by ROS to fluorescent MHR [monohydrorhodamine]) was measured immediately by flow cytometry using an LSR II (BD Biosciences). The data are representative of three experiments with neutrophils obtained from four independent donors. For details on treatment of neutrophils with anti-TLR-2/4 antibodies, please see the supplemental material. Data were analyzed using FlowJo v10.1 (FlowJo, LLC).

### Statistical analyses.

In all cases, results were considered significantly different if *P* < 0.05. However, because of the limited number of samples per group in NHP experiments, we also report *P* values between 0.05 and 0.100, as described previously for NHP experiments (28, 61). All statistical tests were unpaired and two-sided unless mentioned otherwise. For details on all statistical tests used in the study, see the supplemental material.

### Data availability.

All relevant data supporting the key findings of this study are available within the article and its supplemental material or from the corresponding authors upon request.

10.1128/mBio.03115-20.9 TABLE S1 Extracellular and intracellular flow cytometry panels used to evaluate maternal and fetal blood, uterine segments, chorionic villi, and choriodecidual membranes are shown. Download Table S1, DOCX file, 0.02 MB. Copyright © 2021 Coleman et al. 2021 Coleman et al. This content is distributed under the terms of the Creative Commons Attribution 4.0 International license.
