# Supplementary material for: Hyaluronidase Impairs Neutrophil Function and Promotes Group B Streptococcus Invasion and Preterm Labor in Nonhuman Primates
Source: mBio. 2021 Jan 5;12(1):e03115-20. doi: 10.1128/mBio.03115-20 (PMC8545101; doi:10.1128/mBio.03115-20)
Supplement: FIG S1 [file mbio.03115-20-sf001.docx]

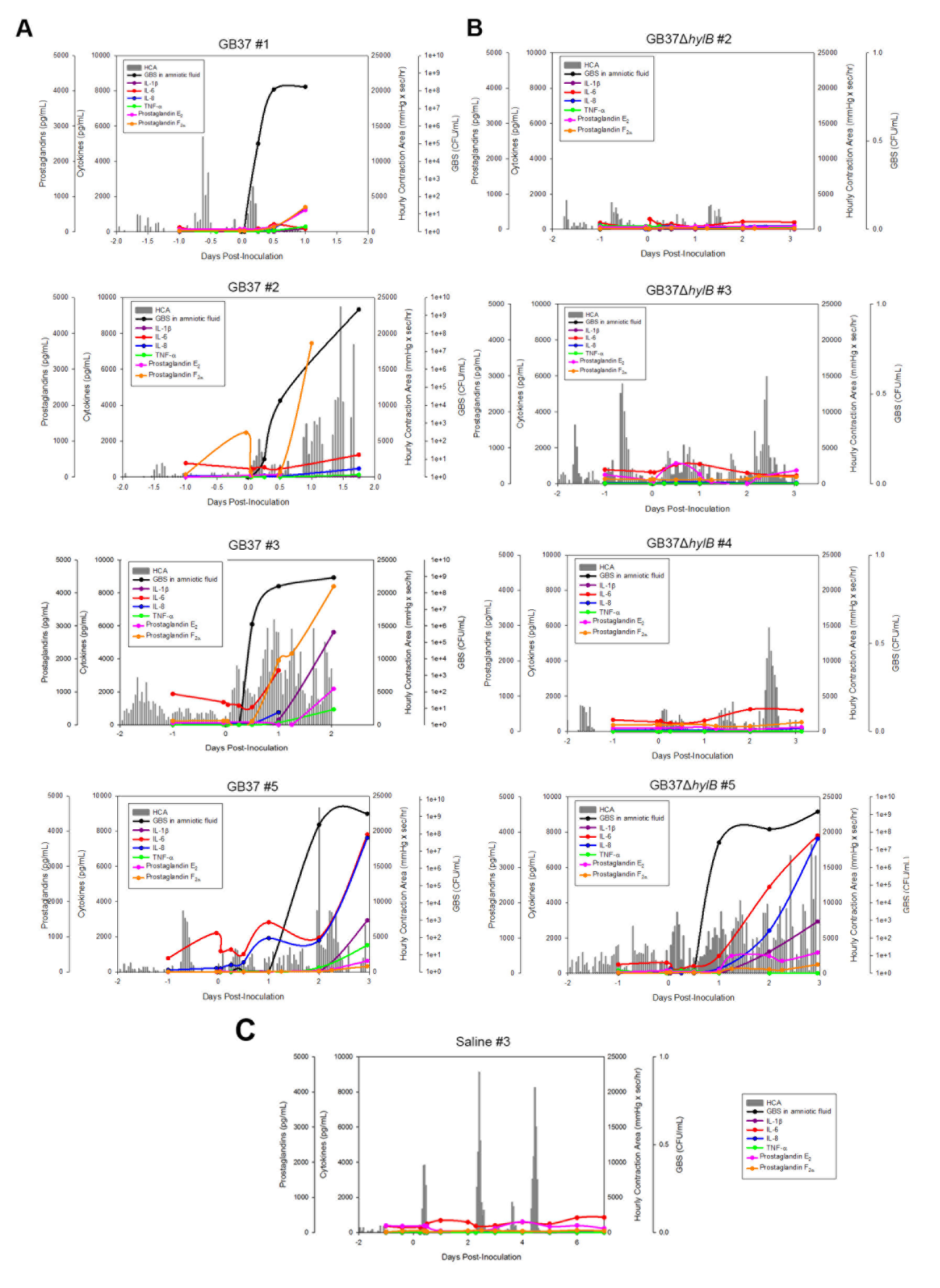


**Supplementary Fig. 1**. Uterine contractions, AF cytokines, prostaglandins, and bacterial CFU from choriodecidual inoculations of GB37, GB37Δ*hylB*, or saline in chronically catheterized pregnant NHP.
