# Supplementary material for: Hyaluronidase Impairs Neutrophil Function and Promotes Group B Streptococcus Invasion and Preterm Labor in Nonhuman Primates
Source: mBio. 2021 Jan 5;12(1):e03115-20. doi: 10.1128/mBio.03115-20 (PMC8545101; doi:10.1128/mBio.03115-20)
Supplement: FIG S3 [file mbio.03115-20-sf003.docx]

**
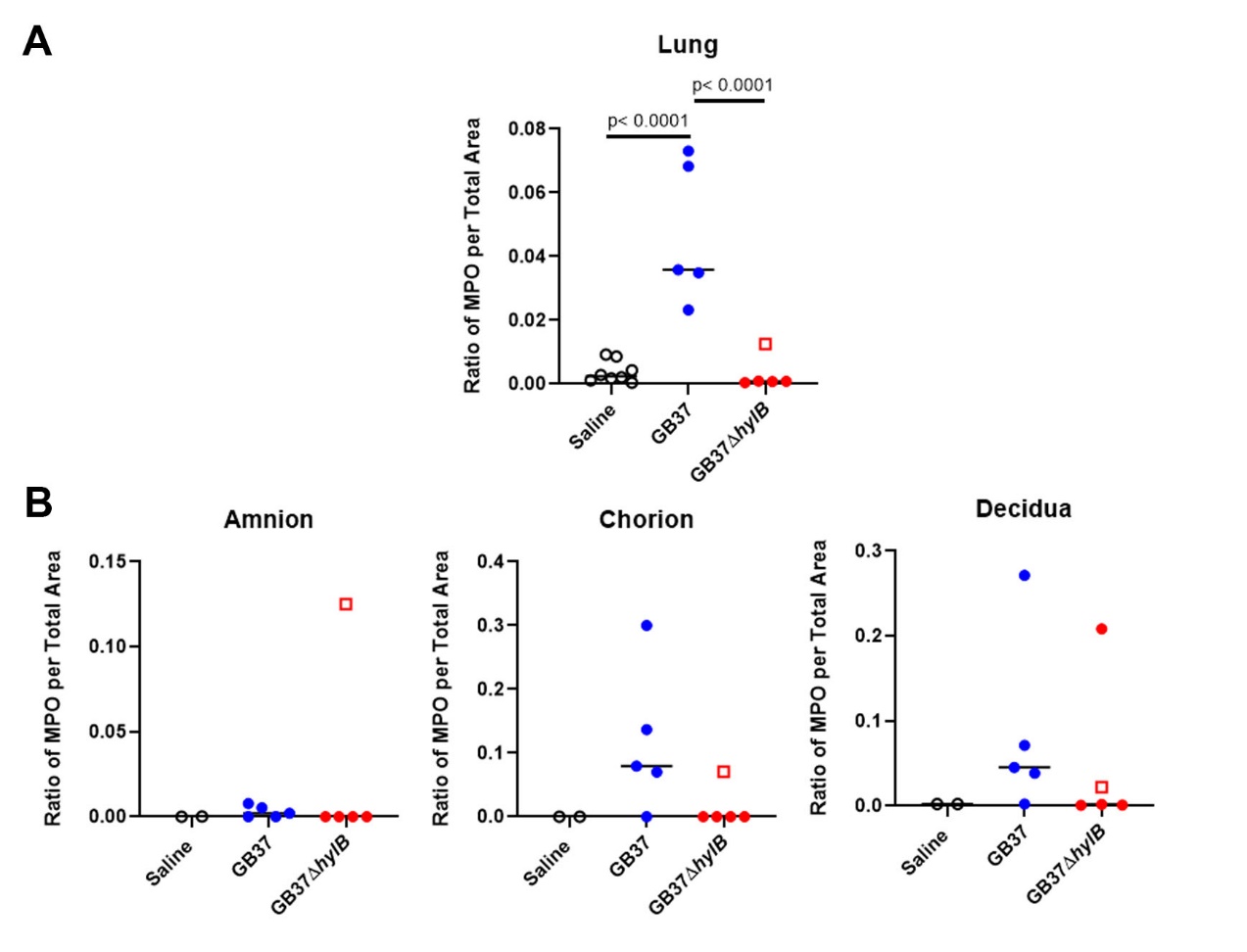
**

**Supplementary Fig. 3**. Quantitation of immunostaining for MPO in the fetal lung and chorioamniotic membranes. (**A**) The area of MPO immunostaining in the neonatal lung was significantly different between Saline and GB37 groups and GB37 and GB37Δ*hylB* groups. GB37Δ*hylB* #5 is designated as an open square. A one-way ANOVA with Tukey’s post-test was used to compare groups. (**B**) The area of MPO immunostaining in the chorioamniotic membranes was not significantly different among the groups in the amnion, chorion, or decidua. GB37Δ*hylB* #5 is designated as an open square. A one-way ANOVA with Tukey’s post-test was used to compare groups.
