# Supplementary material for: Hyaluronidase Impairs Neutrophil Function and Promotes Group B Streptococcus Invasion and Preterm Labor in Nonhuman Primates
Source: mBio. 2021 Jan 5;12(1):e03115-20. doi: 10.1128/mBio.03115-20 (PMC8545101; doi:10.1128/mBio.03115-20)
Supplement: FIG S4 [file mbio.03115-20-sf004.docx]

**
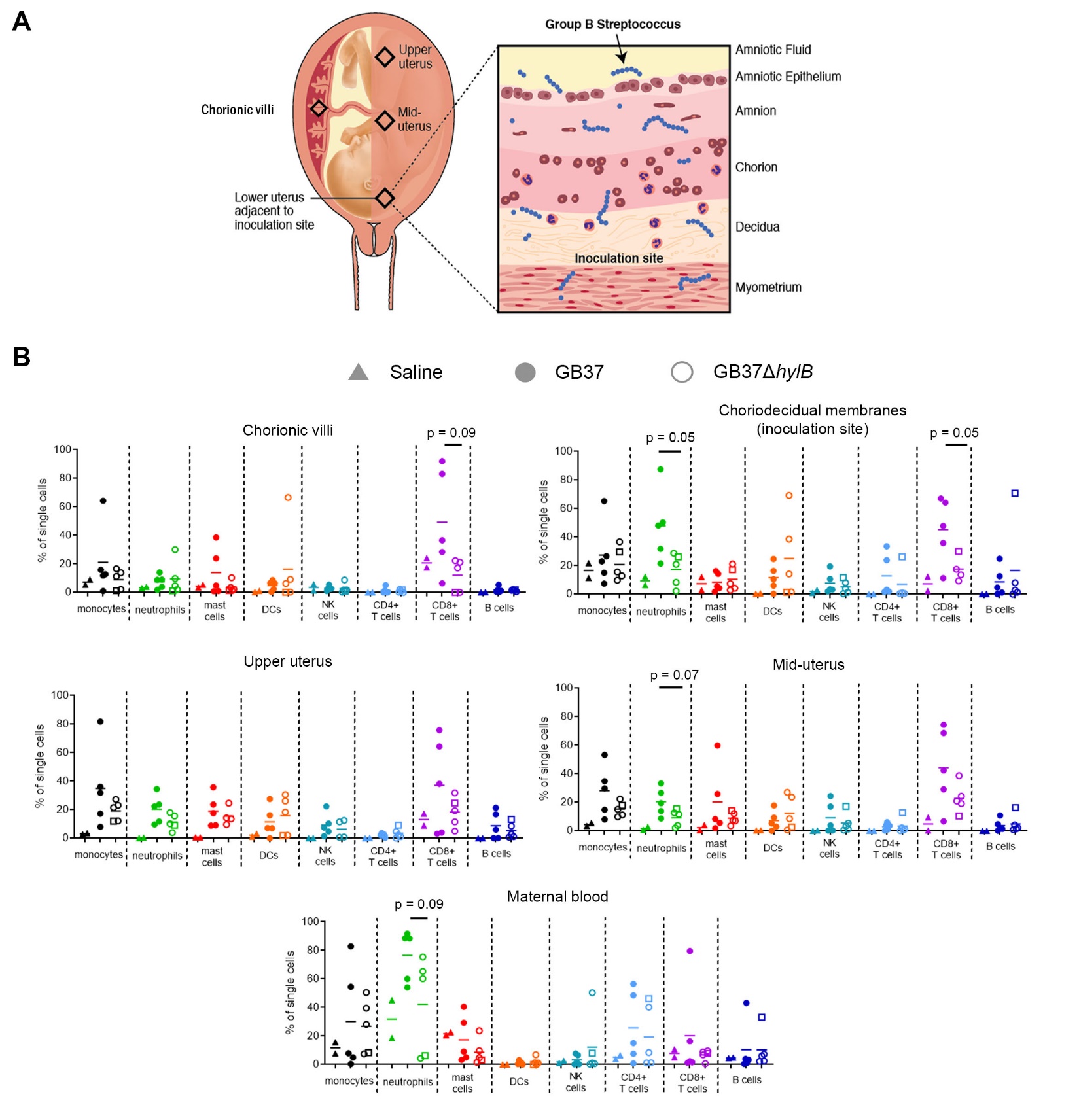
**

**Supplementary Fig. 4**. GB37-inoculated NHP experienced enhanced infiltration of CD8+ T cells and phagocytes to the maternal fetal interface. At Cesarean section, biopsies from the uterus and placenta as well as maternal and fetal blood were obtained. (**A**) A schematic depicting the biopsy sites is shown. (**B**) Samples were processed into single-cell suspensions, stained, and analyzed by flow cytometry for various immune cell markers (indicated in the figure). GB37Δ*hylB* #5 is designated as an open square. A Welch’s test was used to evaluate differences in immune cell populations between GB37- and GB37Δ*hylB*-treated animals at each site. Data from two saline controls performed as a part of the current study are included but similar analyses were not previously performed with historical saline controls (n = 4).
