# Supplementary material for: Hyaluronidase Impairs Neutrophil Function and Promotes Group B Streptococcus Invasion and Preterm Labor in Nonhuman Primates
Source: mBio. 2021 Jan 5;12(1):e03115-20. doi: 10.1128/mBio.03115-20 (PMC8545101; doi:10.1128/mBio.03115-20)
Supplement: FIG S5 [file mbio.03115-20-sf005.docx]

**Supplementary Fig. 5**. Digital Spatial Profiling analyte fold change: GB37 vs.GB37Δ*hylB*. Analyte abundance in distinct placental regions from GB37Δ*hylB*-inoculated NHP and saline-treated NHP were obtained by Digital Spatial Profiling (Nanostring Technologies). Fold changes in analyte abundance (GB37Δ*hylB* over GB37) were log2 transformed and analyzed by a linear mixed model in R version 3.6.2. Significance tests were controlled for false discovery rate.
