# Supplementary material for: Hyaluronidase Impairs Neutrophil Function and Promotes Group B Streptococcus Invasion and Preterm Labor in Nonhuman Primates
Source: mBio. 2021 Jan 5;12(1):e03115-20. doi: 10.1128/mBio.03115-20 (PMC8545101; doi:10.1128/mBio.03115-20)
Supplement: FIG S7 [file mbio.03115-20-sf007.docx]

**Supplementary Fig. 7**. Digital Spatial Profiling analyte fold change: GB37Δ*hylB* vs. saline. Analyte abundance in distinct placental regions from GB37Δ*hylB*-inoculated NHP and saline-treated NHP were obtained by Digital Spatial Profiling (Nanostring Technologies). Fold changes in analyte abundance (GB37Δ*hylB* over saline) were log2 transformed and analyzed by a linear mixed model in R version 3.6.2. Significance tests were controlled for false discovery rate. White asterisk indicates p < 0.05.
