# Supplementary material for: Hyaluronidase Impairs Neutrophil Function and Promotes Group B Streptococcus Invasion and Preterm Labor in Nonhuman Primates
Source: mBio. 2021 Jan 5;12(1):e03115-20. doi: 10.1128/mBio.03115-20 (PMC8545101; doi:10.1128/mBio.03115-20)
Supplement: FIG S8 [file mbio.03115-20-sf008.docx]

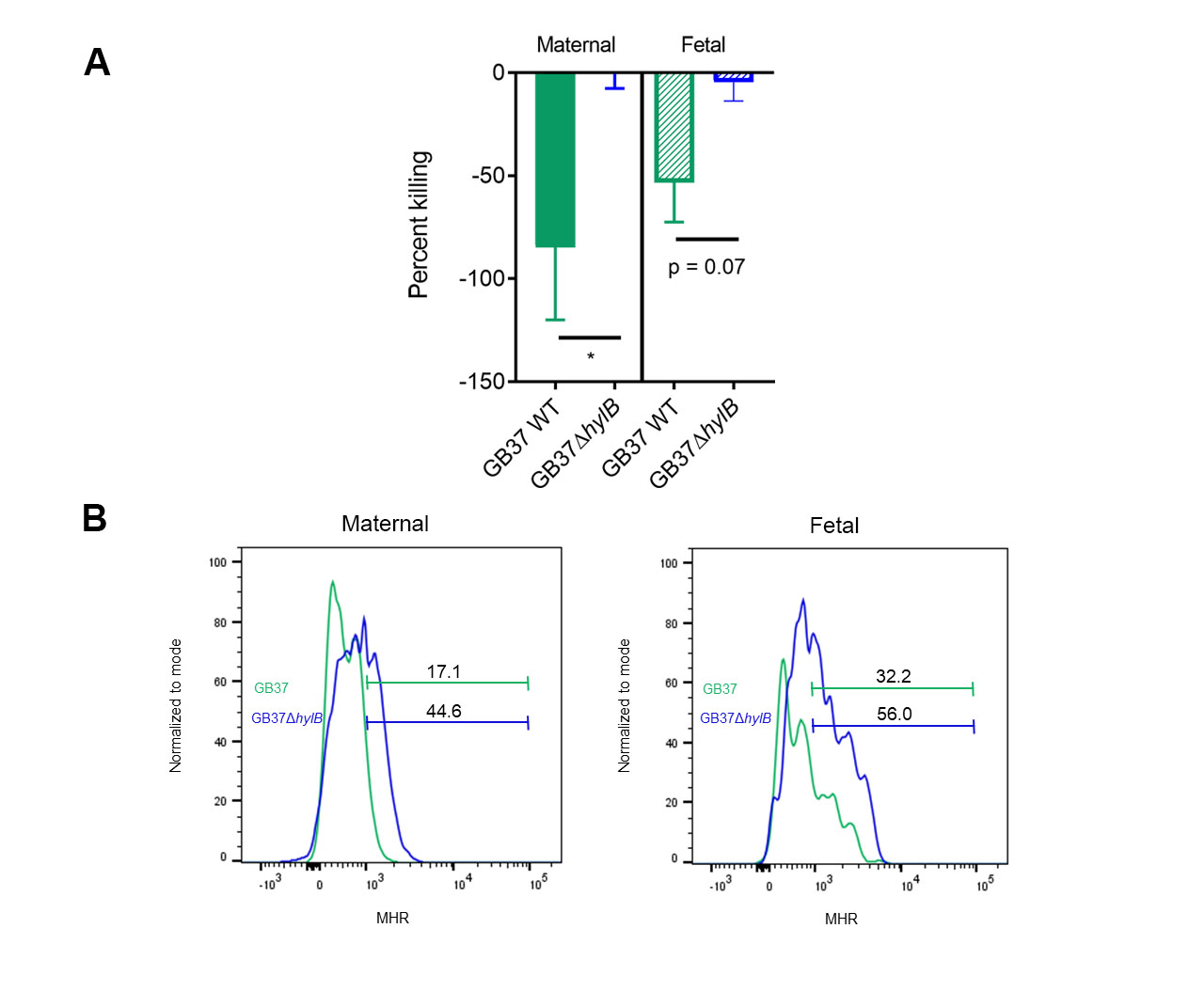


**Supplementary Fig. 8.** Neutrophils were isolated from human maternal blood and umbilical cord (i.e. fetal) blood. (**A**) GB37 or GB37Δ*hylB* were exposed to maternal or fetal neutrophils (MOI 1) for one hour. Percent killing was calculated as the number of CFU recovered after incubation with neutrophils out of the number of the number CFU recovered after incubation without neutrophils (x 100). Differences among groups were determined using a paired t test. * indicates p < 0.05. (**B**) Maternal or fetal neutrophils were pre-treated with dihydrorhodamine 123 (DHR), and then exposed to GB37 (green) or GB37Δ*hylB* (blue) (MOI 100). The conversion of DHR to fluorescent MHR indicates ROS production in cells and was measured by flow cytometry at 60 minutes post-infection.
