# Supplementary material for: Hyaluronidase Impairs Neutrophil Function and Promotes Group B Streptococcus Invasion and Preterm Labor in Nonhuman Primates
Source: mBio. 2021 Jan 5;12(1):e03115-20. doi: 10.1128/mBio.03115-20 (PMC8545101; doi:10.1128/mBio.03115-20)
Supplement: TEXT S1 [file mbio.03115-20-s0001.docx]

**Supplemental Methods**

Methods

*Study design*

NHP animals: A sample size of n = 5 was predefined (prior to beginning of study) to provide an estimated 80% power in achieving statistically significant results in comparisons among and between animals in each treatment group (ANOVA with alpha level of 0.025). Two-sided unpaired tests were used in the final analyses.

Rationale and design of study: The objective of this study was to determine if expression of hyaluronidase by GBS promoted GBS invasion of the amniotic fluid and fetus and/or preterm labor (PTL). Ten animals received choriodecidual inoculations of 1-3 x 10^8^ colony forming units (CFU) of either hyaluronidase-expressing GBS (GB37) or an isogenic hyaluronidase deficient strain (GB37Δ*hylB*). The results from each treatment group were compared to one another and to saline controls (n = 6), four of which were previously described ^27^. The primary outcomes of this study are shown in Table 1. Of note, the primary outcome, denoted as “adverse outcome,” was a composite of preterm labor and/or microbial invasion of the amniotic cavity, as both are poor outcomes of pregnancy.

*Generation of GB37ΔcpsE*

The isogenic strain GB37Δ*cpsE* was derived from wild-type GB37 using methods described previously ^22, 72^. Briefly, approximately 1.0-kb of DNA flanking either side of the *cpsE* gene was PCR amplified using GB37 genomic DNA as template and the primers (for 1KB upstream: 5’-ATTTAAAGATACCCCCAATACAAGAGCCCCTTACTTCC-3’ and 5’-GGGGCTCTTGTATTGGGGGTATCTTTAAATACTG3'; for 1KB downstream: 5’-AAAGGATCCAATCCAAAAATGTCTCAAAAATTAG-3' and 5’-AAAAAGCTTTCCTCCTATTAACAAATAC3').  The region encoding kanamycin resistance was PCR amplified from the plasmid pCIV2 ^73^ using primers (5’-CAAATTTTCATTATACTAAAACAATTCATCCAG-3’ and  5’-GGATGAATTGTTTTAGTATAATGAAAATTTGTCTGG-3'). All PCR fragments were ligated using the Gibson Assembly cloning kit (New England Biolabs) into the linearized temperature-sensitive vector pHY304 ^74^. The resulting plasmid was then electroporated into electrocompetent GB37, and selection for the double crossover mutant with an allelic replacement of *cpsE* with the gene conferring kanamycin resistance was performed as previously described ^72^.

*Catheterization of pregnant NHP*

Between days 116 and 125 of pregnancy (term = 172 days), NHP were catheterized by laparotomic surgical implantation into the maternal femoral vein, amniotic cavity, and choriodecidual interface in the lower uterine segment (i.e., between the uterine muscle and fetal membranes, external to amniotic cavity) as described ^27, 30, 75^. Choriodecidual and amniotic fluid catheters were made of polyvinyl tubing, and maternal artery and vein catheters were made of silicone tubing. A maternal temperature probe (AD Instruments) was also implanted. Post-operative analgesia was provided via a fentanyl patch applied one day prior to surgery, in addition to post-operative indomethacin or ketoprofen as described ^27, 30^.

Following surgery, each animal was placed in the jacket and tether with the catheters/electrodes tracked through the tether system. Cefazolin and terbutaline sulfate were administered intravenously to reduce postoperative infection risk and uterine activity. Cefazolin and terbutaline were stopped at least 72 hours prior to experimental start (97% of drugs eliminated) and represented approximately a 7- to 10-day period of postoperative terbutaline administration. To allow for recovery, experiments began approximately two weeks after catheterization surgery (equivalent to approximately 30 to 31 weeks human gestation).

*NHP sample collection*

AF (~4 mL) and maternal blood (~3 mL, in EDTA tubes, BD Biosciences) were sampled before (at -24 and -0.25 hours) and after inoculation (0.75, +6, +12, +24 hours and then every 12 hours until Cesarean section for fetal necropsy). To quantify bacterial invasion of the amniotic cavity, AF (200 μL) from each sampling was serially diluted by a factor of 10, and dilutions were plated on TSA, incubated overnight at 37 °C, 5% CO_2_, and GBS CFU were enumerated. For cytokine prostaglandin (PG), and matrix metalloprotease (MMP) analysis, samples of AF and material blood were centrifuged for 5 min at 300 x *g* immediately after collection, and indomethacin (0.3 mM) was added to supernatants to halt PG metabolism prior to freezing at -80 °C.

Fetal blood was obtained during Cesarean section, and fetal tissues were obtained and weighed during necropsy. To analyze bacterial dissemination into fetal organs, fetal tissues were homogenized in sterile PBS on ice using a hand-held tissue homogenizer (Tissue Tearor, Biospec Products, Inc.) in 10 to 30 second pulses until the samples were completely homogenous. Subsequently, 10-fold serial dilutions were plated on TSA and incubated overnight at 37 °C, 5% CO_2_, and GBS CFU were enumerated, as described ^77^.

*Measuring cytokines and MMP*

To analyze fetal inflammatory cytokines, fetal organ homogenates were diluted 1:1 in lysis buffer (150 mM NaCl, 15 mM Tris, 1 mM MgCl2, 1 mM CaCl2, 1% Triton X-100, supplemented with cOmplete, Mini, EDTA-free protease inhibitor cocktail (Roche)) and incubated overnight at 4 °C. Then, lysates were centrifuged at 600 x *g* for 5 min at 4 °C, and supernatants were analyzed immediately or stored at -80 °C for later analysis. Cytokine (IL-1β, TNF-α, IL-6, and IL-8) levels were determined using ProcartaPlex cytokine kits (ThermoFisher), per manufacturer instructions. Prostaglandin E2 (PGE2) and prostaglandin F2-alpha (PGF2α) were determined using commercially available ELISA kits (Cayman Chemical), per manufacturer instructions. AF and lysates from homogenized lower uterine biopsies (processed as above) were analyzed for MMP levels using a human MMP human LXSAHM-06 kit (R&D Systems) according to manufacturer instructions.

*Flow cytometry of placental tissues, uterine tissues, maternal blood, and fetal blood*

Intact placental segments, including chorioamniotic membranes and chorionic villi (CV), as well as biopsies from the myometrium (lower uterus, mid-uterus, and upper uterus) were obtained during Cesarean section and stored in RPMI 1640 with L-glutamine (Corning, hereafter referred to as RPMI-g) on ice for approximately 30 min prior to tissue processing. Maternal and fetal blood (approx. 1 mL) were also collected during Cesarean section and stored in 1X red blood cell (RBC) lysis buffer (150 mM NH_4_Cl, 0.1 mM NaHCO_3_, 1.27 mM EDTA in distilled, deionized H_2_O) on ice.

Placental and uterine tissues were washed twice with PBS, then weighed. Digestion buffer (20 mM HEPES, 30 mM NaHCO_3_, 150 µg/mL DNase I (Roche), 1 mg/mL hyaluronidase from bovine testes, 10 mg/mL BSA, 0.75 mg/mL Collagenase A (Roche), 100 µg/mL streptomycin, 100 U/mL penicillin in RPMI-g) was added to each tissue at 10 mL/g tissue, and then each tissue was minced to approximately 1 cm^3^, transferred to a 50 mL conical tube, and incubated at 37 °C for 1 hour while shaking at 300 rpm. During the incubation, blood samples were washed 3 to 5 times in 1X RBC lysis buffer (until RBCs were visibly absent from cell pellets), resuspended in RPMI-g supplemented with 10% heat-inactivated fetal bovine serum (Gibco) and 5% penicillin/streptomycin (hereafter referred to as RPMI-g + FBS + p/s) and stored at 4 °C. Following digestion, macerated placental and uterine tissues were washed once (300 x *g*, 5 minutes), resuspended in RPMI-g + FBS + p/s, and then progressively passed through a 280 μm metal sieve and a 40 μm nylon screen. Single cells were pelleted and resuspended in RPMI-g + FBS + p/s. Cell suspensions from all tissues and blood were counted using a TC20 cell counter (BioRad), diluted to approximately 1.5 x 10^7^ cells/mL in RPMI-g + FBS + p/s, and stored at 4 °C overnight.

Cell suspensions were transferred to wells of a 96-well plate (approximately 3 x 10^6^ cells/well), washed once with FACS buffer (300 x *g*, 5 minutes), and then incubated in 100 μL human Fc block (1:200, BD Biosciences) for 15 min at room temperature. Cells were then resuspended in 100 μL of the appropriate extracellular panel (Supplementary Table 1) and incubated for 15 to 25 minutes at room temperature, protected from light. Cells were washed twice in FACS buffer, then resuspended in 200 μL fixation/permeabilization working solution from the Intracellular Fixation & Permeabilization Buffer Set (eBioscience). After incubating for 30 minutes at room temperature, protected from light, cells were washed twice in fixation/permeabilization buffer (Fixation & Permeabilization Buffer Set, eBioscience) and then resuspended in 100 μL of the appropriate intracellular panel (Supplementary Table 1). Cells stained for 30-35 minutes at room temperature, protected from light, and were washed twice in fixation/permeabilization buffer, resuspended in 100 μL FACS buffer, and aliquoted into FACS tubes containing 700 μL FACS buffer. Cells were analyzed for intracellular and extracellular markers using an LSRII flow cytometer (BD Biosciences), and unstained cells and single-stained compensation beads (BD Biosciences) were used for compensation. Data were analyzed using FlowJo software version 10.1 (FlowJo).

*MPO and CD68 immunostaining*

The University of Washington Histology and Imaging Core performed the immunohistochemistry optimization and staining for MPO and CD68. We used the following primary, secondary and tertiary antibodies: rabbit polyclonal MPO (Clone Ab-1, ThermoScientific, Catalog Number RB-373-A1), mouse monoclonal CD68 (Clone 514H12, Leica, Catalog Number PA0273), Leica Post-Primary linker and goat anti-rabbit horseradish peroxidase polymerized antibody (Leica Catalog Number DS9800). Utilizing the Leica Bond Rx Automated Immunostainer (Leica Microsystems, Buffalo Grove, IL), slides were first deparaffinized with Leica Dewax Solution at 72 °C for 30 seconds. For MPO staining, antigen retrieval was heat-mediated using citrate, pH 6, at 100 °C for 20 minutes. For CD68 staining, antigen retrieval was performed with EDTA, pH 9, at 100 °C for 20 minutes. All subsequent steps were at room temperature. Blocking was performed with 10% normal goat serum (Jackson ImmunoResearch, Catalog Number 005-000-121) in tris-buffered saline for 20 minutes followed by blocking with Leica Bond Peroxide Block for 5 minutes. Slides were then incubated with either the MPO (1:100) primary antibody in Leica Primary Antibody Diluent or CD68 (no dilution) primary antibody for 30 minutes. For MPO, a secondary antibody (goat anti-rabbit horseradish peroxidase polymerized antibody) was applied for 8 minutes. For CD68, the Leica Post-Primary linker was applied for 8 minutes and tissues were then incubated with a tertiary antibody (goat anti-rabbit horseradish peroxidase polymerized antibody) for 8 minutes. Antibody complexes were visualized using DAB (3,3’-diaminobenzidine), detection 2X for 10 minutes. Tissues were counterstained with hematoxylin. Slides were removed from the automated stainer, dehydrated and coverslipped. Unless otherwise specified, all reagents were obtained from Leica Microsystems. Quantitative imaging was the performed using Visiopharm image analysis software (Visiopharm, Inc., Hoersholm, Denmark). Regions of interest within each tissue were outlined corresponding to the amnion, chorion and decidua within the chorioamniotic membranes and alveoli within the fetal lung. The ratio of the immunostained area of tissue to the area of the entire tissue within the region of interest was calculated and compared across groups using one-way ANOVA with adjustment for multiple comparisons using Tukey’s test.

*Isolation of neutrophils from adult human blood*

As described ^30^, 5-15 mL of human blood was collected independently from healthy human adults into EDTA tubes (BD Biosciences). Immediately following collection, neutrophils were isolated using a MACSxpress neutrophil isolation kit, per the manufacturer’s instructions (Miltenyi Biotec). Cells were then pelleted, and any residual red blood cells (RBC) were removed by resuspending the cell pellet in RBC lysis buffer for 15 minutes at room temperature. After RBC lysis, cells were washed with RPMI-g. Neutrophil purity in the prepared cell suspension was assessed by examining the proportion of cells positive for the neutrophil cell markers CD15 (PerCP/Cy5.5, clone HI98, BD Biosciences) and CD16 (FITC, clone 3G8, BD Biosciences) by flow cytometry. Briefly, approximately 1 x 10^6^ cells from the neutrophil purification preparation or 1 x 10^6^ cells from whole blood (following two RBC lysis steps, as described above) were incubated with human Fc block (1:200, BD Biosciences) for 15 minutes at room temperature. Then, immunofluorescent antibodies were added to the cells at concentrations recommended by the manufacturer (1:10, CD15-PerCP/Cy5.5; 1:200, CD16-FITC), and cells incubated for 30 minutes at room temperature. Stained cells were washed twice in FACS buffer and were analyzed immediately on an LSR II flow cytometer (BD Biosciences). Single-stained flurochrome-reactive AbC beads and unstained cells were used for compensation. Data were analyzed using FlowJo v. 10.1 (FlowJo, LLC).

*Isolation of neutrophils from maternal and cord blood*

Neutrophils were also isolated from maternal and cord blood using methods described above. Women enrolled in this study provided informed consent as previously described and underwent elective Cesarean sections at term in the absence of labor. Maternal blood was obtained prior to Cesarean section.

*Neutrophil killing assay*

Neutrophils were isolated from fresh human blood as indicated above and washed with RPMI-g. Then, approximately 1 x 10^6^ neutrophils were incubated with GB37 or GB37Δ*hylB* at MOI 1 in RPMI-g for 1 hour at 37 °C as described ^30, 78^. Triton X-100 (0.1%) was added to lyse neutrophils and release intracellular bacteria and total bacteria (intracellular and extracellular) were enumerated by serial dilution plating on TSA. Percent killing was calculated as the number of CFU recovered in the presence of neutrophils over the number of CFU recovered in the absence of neutrophils (x 100).

*Measurement of ROS production by neutrophils*

Human neutrophils were resuspended in HBSS at 1 x 10^6^ cells/mL and pre-incubated with 84 μM dihydrorhodamine 123 (DHR in 0.28% DMSO) at 37 °C for 20 minutes, as described ^30^. Approximately 5 x 10^5^ pre-treated neutrophils were exposed to GB37, GB37Δ*hylB*, or GB37Δ*cpsE* (MOI 100) for 60 minutes, and the fluorescence intensity of cells (which measures DHR oxidation by ROS to fluorescent MHR) was measured immediately by flow cytometry using an LSR II (BD Biosciences). Data are representative of 2 experiments with neutrophils obtained from independent donors and were analyzed using FlowJo v. 10.1 (FlowJo, LLC).

*Measuring the effect of TLR-2/4 signaling on ROS production by neutrophils*

Filtered supernatants of stationary phase GB37 or GB37Δ*hylB* liquid cultures were incubated with HA for 18 hours to allow for enzymatic digestion of HA. Primary human neutrophils were resuspended to 1 x 10^7^ cells/mL and pre-treated with 10 μg/mL anti-TLR-2 antibody (Invivogen) plus 10 μg/mL anti-TLR-4 antibody (Invivogen) or vehicle control for 15 minutes at room temperature. Then, neutrophils were diluted to 1 x 10^6^ cells/mL in HBSS and were treated with 84 μM dihydrorhodamine 123 (DHR) at 37 °C for 20 minutes. Neutrophils were then exposed to the digested HA solutions from each strain for 60 minutes, and ROS production in cells was measured by detecting fluorescent MHR via flow cytometry as above (LSR II, BD Biosciences). Data were analyzed with FlowJo v. 10.1 (FlowJo, LLC).

*Statistical analyses*

For *in vivo* studies (NHP experiments) the primary study outcomes were compared using Barnard’s test. Changes in peak AF cytokines and prostaglandins were compared using an analysis of one-way ANOVA with Bonferroni’s correction. These analyses were performed using Intercooled STATA 8.2 for Windows (StatCorp) or SciStatCalc. CFU in fetal tissues were compared using the Mann-Whitney test, as normal distribution was not assumed in these samples. Similarly, cytokine levels in fetal tissues were not assumed to have equal distribution and were thus compared using test Kruskal-Wallis test with Dunn’s multiple comparison test. Welch’s test was used to compare immune cell populations detected by flow cytometry in GB37 vs. GB37Δ*hylB* animals. A mixed linear model controlling for false discovery rate was used to compare differences in DSP analyte abundance among GB37, GB37Δ*hylB*, and saline. In all cases, results were considered significantly different if p < 0.05. However, because of the limited number of samples per group in NHP experiments, we also report trends (i.e. p values between 0.05 and 0.100) as described previously for NHP experiments ^30, 79^. For *in vitro* experiments with greater than two comparison groups, Kruskal-Wallis test or one-way ANOVA was performed with Dunn’s or Tukey’s post-test, respectively, and as indicated in figure legends. For *in vitro* experiments with two comparison groups, a student’s t test was performed. All statistical tests were unpaired and two-sided unless mentioned otherwise. These analyses were performed using GraphPad Prism version 6.0 (GraphPad Software, [www.graphpad.com](http://www.graphpad.com)), unless otherwise noted.
