# Supplementary material for: Hyaluronidase Impairs Neutrophil Function and Promotes Group B Streptococcus Invasion and Preterm Labor in Nonhuman Primates
Source: mBio. 2021 Jan 5;12(1):e03115-20. doi: 10.1128/mBio.03115-20 (PMC8545101; doi:10.1128/mBio.03115-20)
Supplement: TABLE S1 [file mbio.03115-20-st001.docx]

**Supplementary** **Table 1**. Extracellular and intracellular flow cytometry panels used to evaluate maternal and fetal blood, uterine segments, chorionic villi, and choriodecidual membranes are shown.

| **Extracellular flow cytometry panels** | | | | | | | | | | |
| --- | --- | --- | --- | --- | --- | --- | --- | --- | --- | --- |
| **Panel 1** | **Marker** | | **Clone** | | **Color** | | **Manufacturer (cat #)** | | **Dilution** | |
| T cells | CD3 | | SP34 | | AF700 | | BD (357917) | | 1:7 | |
|  | CD4 | | L200 | | FITC | | BD (550628) | | 1:7 | |
|  | CD8 | | SK1 | | APC-Cy7 | | BioLegend (344714) | | 1:10 | |
| **Panel 2** | **Marker** | | **Clone** | | **Color** | | **Manufacturer (cat #)** | | **Dilution** | |
| B cells, DCs | CD45 | | D085-1283 | | AF700 | | BD (561288) | | 1:10 | |
|  | CD20 | | L27 | | PE | | BD (346595) | | 1:5 | |
|  | CD209 | | D CN46 | | PerCP/Cy5.5 | | BD (558263) | | 1:5 | |
|  | HLA-DR | | L243 | | FITC | | BD (347363) | | 1:5 | |
| **Panel 3** | **Marker** | | **Clone** | | **Color** | | **Manufacturer (cat #)** | | **Dilution** | |
| macrophages, neutrophils | CD45 | | D085-1283 | | AF700 | | BD (561288) | | 1:10 | |
|  | CD14 | | M5E2 | | BV421 | | BD (565283) | | 1:5 | |
|  | CD11c | | S-HCL-3 | | PE | | BD (347637) | | 1:5 | |
|  | CD66abce | | Tet2 | | FITC | | Miltenyi Biotec (130-093-132) | | 1:5 | |
| **Panel 4** | **Marker** | | **Clone** | | **Color** | | **Manufacturer (cat #)** | | **Dilution** | |
| NK cells, mast cells | CD45 | | D085-1283 | | AF700 | | BD (561288) | | 1:10 | |
|  | CD49d | | 9F10 | | BV421 | | BD (565277) | | 1:20 | |
|  | NKG2a | | REA110 | | PE | | Miltenyi Biotec (130-098-814) | | 1:10 | |
| **Intracellular flow cytometry panels** | | | | | | | | | | |
| **Panel 1** | | **Marker** | | **Clone** | | **Color** | | **Manufacturer (cat #)** | | **Dilution** |
| T cells | | IL-4 | | MP4-25D2 | | APC | | BD (554486) | | 1:20 |
|  |  | IL-17 | | eBio64DEC17 | | PerCP/Cy5.5 | | Invitrogen (45-7179-42) | | 1:10 |
|  |  | IFN-g | | B27 | | PE/Cy7 | | BD (557643) | | 1:20 |
|  |  | Ki67 | | B56 | | BV421 | | BD (562899) | | 1:20 |
|  |  | FoxP3 | | 206D | | PE | | BioLegend (320108) | | 1:10 |
| **Panel 2** | | **Marker** | | **Clone** | | **Color** | | **Manufacturer (cat #)** | | **Dilution** |
| B cells, DCs | | IL-4 | | MP4-25D2 | | APC | | BD (554486) | | 1:20 |
|  |  | Ki67 | | B56 | | BV421 | | BD (562899) | | 1:20 |
| **Panel 3** | | **Marker** | | **Clone** | | **Color** | | **Manufacturer (cat #)** | | **Dilution** |
| macrophages, neutrophils | | Granzyme B | | GB12 | | APC | | Invitrogen (MHGB05) | | 1:20 |
| **Panel 4** | | **Marker** | | **Clone** | | **Color** | | **Manufacturer (cat #)** | | **Dilution** |
| NK cells, mast cells | | Granzyme B | | GB12 | | APC | | Invitrogen (MHGB05) | | 1:20 |
