# Supplementary material for: Hyaluronidase Impairs Neutrophil Function and Promotes Group B Streptococcus Invasion and Preterm Labor in Nonhuman Primates
Source: mBio. 2021 Jan 5;12(1):e03115-20. doi: 10.1128/mBio.03115-20 (PMC8545101; doi:10.1128/mBio.03115-20)
Supplement: FIG S2 [file mbio.03115-20-sf002.docx]

**Supplementary Fig. 2**. Histological examination of NHP fetal lung sections. (**A-D**) Representative H&E stained sections from NHP in each group are shown, including Saline #3 (**A**), GB37 # 1 (**B**), GB37 # 2 (**C**), and GB37Δ*hylB* #2 (**D**). (**E-H**) Representative MPO stained sections from NHP in each group are shown, including Saline #3 (**E**), GB37 #1 (**F**), GB37 #2 (**G**), and GB37Δ*hylB* #2 (**H**). (**I-L**) Representative CD68 stained sections from NHP in each group are shown, including Saline #3 (**I**), GB37 #1 (**J**), GB37 #1 (**K**), and GB37Δ*hylB*  #2 (**L**). Note that within the GB37 group, tissues are shown reflecting harvest at 24 hours (B, F, J) and 48 hours (C, G, K).
